# An Inducer of VGF Protects Cells against ER Stress-Induced Cell Death and Prolongs Survival in the Mutant SOD1 Animal Models of Familial ALS

**DOI:** 10.1371/journal.pone.0015307

**Published:** 2010-12-09

**Authors:** Masamitsu Shimazawa, Hirotaka Tanaka, Yasushi Ito, Nobutaka Morimoto, Kazuhiro Tsuruma, Michinori Kadokura, Shigeki Tamura, Teruyoshi Inoue, Mitsunori Yamada, Hitoshi Takahashi, Hitoshi Warita, Masashi Aoki, Hideaki Hara

**Affiliations:** 1 Molecular Pharmacology, Department of Biofunctional Evaluation, Gifu Pharmaceutical University, Gifu, Japan; 2 Biomedical Research Laboratories, Asubio Pharma Co., Ltd., Osaka, Japan; 3 Department of Clinical Research, National Hospital Organization, Saigata National Hospital, Niigata, Japan; 4 Department of Pathology, Brain Research Institute, Niigata University, Niigata, Japan; 5 Department of Neurology, Tohoku University School of Medicine, Sendai, Japan; Universidade Federal do Rio de Janeiro, Brazil

## Abstract

Amyotrophic lateral sclerosis (ALS) is the most frequent adult-onset motor neuron disease, and recent evidence has suggested that endoplasmic reticulum (ER) stress signaling is involved in the pathogenesis of ALS. Here we identified a small molecule, SUN N8075, which has a marked protective effect on ER stress-induced cell death, in an *in vitro* cell-based screening, and its protective mechanism was mediated by an induction of VGF nerve growth factor inducible (VGF): VGF knockdown with siRNA completely abolished the protective effect of SUN N8075 against ER-induced cell death, and overexpression of VGF inhibited ER-stress-induced cell death. VGF level was lower in the spinal cords of sporadic ALS patients than in the control patients. Furthermore, SUN N8075 slowed disease progression and prolonged survival in mutant SOD1 transgenic mouse and rat models of ALS, preventing the decrease of VGF expression in the spinal cords of ALS mice. These data suggest that VGF plays a critical role in motor neuron survival and may be a potential new therapeutic target for ALS, and SUN N8075 may become a potential therapeutic candidate for treatment of ALS.

## Introduction

**Figure 1:**
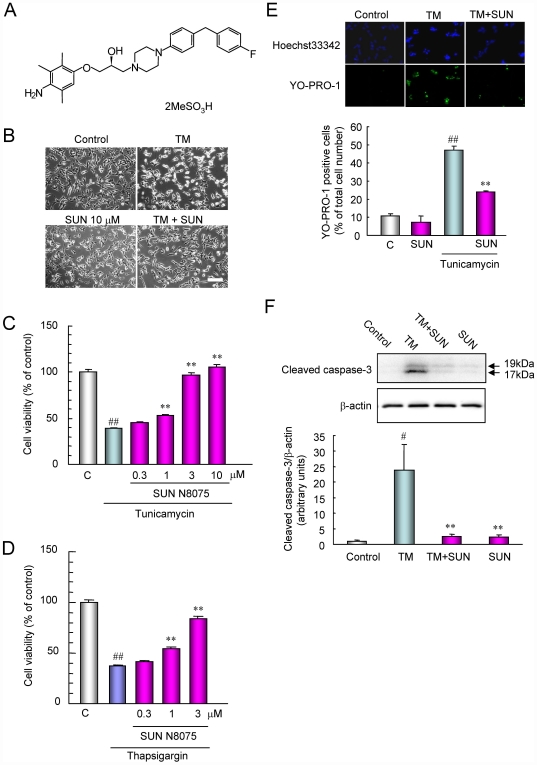
SUN N8075 protects against ER stress-induced cell death and attenuates cleaved caspase-3 production. (A) Chemical structure of SUN N8075, (B) Representative phase-contrast microscopy showing the effect of SUN N8075 on morphological changes in SH-SY5Y cells after tunicamycin at 2 µg/ml. Bar  = 50 µm. (C, D) Cell damage was induced by tunicamycin (2 µg/ml) or thapsigargin (2 µM), and cell viability was measured with the tetrazolium salt (WST-8) reduction test. SUN N7085 or vehicle was applied 1 h before the tunicamycin or thapsigargin treatment. Each column represents the mean ± S.E.M. (*n* = 8). ##*p*<0.01 versus control. **p<0.01 versus tunicamycin or thapsigargin alone. (E) Representative fluorescence microscopy showing nuclear stainings for Hoechst 33342 (blue) and YO-PRO-1 (green) at 24 h after the addition of 2 µg/ml of tunicamycin with or without SUN N8075. Each column represents the mean ± S.E.M. (n = 6). ** and ##*p*<0.01 versus relevant control group. (F) Tunicamycin increased cleaved caspase-3, and treatment with SUN N8075 inhibited the increase of cleaved caspase-3 after tunicamycin. Each column represents the mean ± S.E.M. (*n* = 6). #*p*<0.05 versus control. **p<0.01 versus tunicamycin alone.

In chronic neurodegenerative disorders such as Alzheimer's disease, Parkinson's disease, Huntington's disease (HD), and amyotrophic lateral sclerosis (ALS), abnormally unfolded proteins are known to aggregate and accumulate in neurons, and these proteins are thought to be closely related to the initiation and development of these neurodegenerative diseases [1], [2], [3]. Recent studies suggest that endoplasmic reticulum (ER) stress plays a role in the pathogenesis of familial and sporadic ALS [4], [5], [6]. A variety of conditions such as environmental and genetic causes that cause unfolded or misfolded proteins in the ER can activate the ER stress response although the cell normally survives the insult. However, excessive or prolonged ER stress can induce cell death, usually in the form of apoptosis. In familial ALS model mice carrying the mutant SOD1 gene, mutant superoxide dismutase-1 forms aggregates in the ER have been reported to induce the expression of 78 kDa glucose-regulated protein (GRP78/BiP), an ER resident molecular chaperone, and to activate caspase-12, leading to neuronal cell death [7], [8]. Furthermore, Ilieva et al. [6] have reported increased ER chaperones such as protein-disulfide isomerase (PDI) and phosphorylation of eukaryotic initiation factor 2α (eIF2α) in spinal cords from patients with sporadic ALS. Thus, although the etiologies such as the responsible genes or environmental factors are different for familial and sporadic ALS, they may have a common mechanism for neuronal cell death through ER stress as downstream signaling pathways. Despite many clinical trials for treatment of ALS, there has been little success in the search for neuroprotective agents. Therefore, we screened for protecting cells from ER stress, and identified a small molecule, SUN N8075 (Fig. 1A). SUN N8075 has been reported to have a potent antioxidant property [9], and is currently in a phase I clinical trial for stroke. In the present study, we demonstrated that SUN N8075 induces VGF nerve growth factor inducible (VGF) as a mechanism for protecting ER stress-induced cell death in an antioxidant-independent manner. VGF is a neuronal polypeptide first identified as a cDNA clone from plate V of the nerve growth factor (NGF)-induced rat pheochromocytoma (PC12) cell cDNA library [10]. VGF is widely expressed in neurons in the brain and involved in maintaining organism energy balance, as well as in mediating hippocampal synaptic activity [11], [12]. Interestingly, a recent study reported that VGF content was decreased in the cerebrospinal fluid (CSF) of ALS patients and in the serum, CSF and spinal cord motor neurons of G93A mice [13]. These findings strongly suggest that a VGF inducer, SUN N8075, may become a potential therapeutic candidate for ALS. In the present study, we demonstrated that (i) VGF is involved in the protective effects of SUN N8075 on ER stress-induced cell death and (ii) SUN N8075 protects against disease progression and prolongs survival in familial ALS models involving ER stress.

## Materials and Methods

### Chemicals

(2S)-1-(4-Amino-2,3,5-trimethylphenoxy)-3-{4-[4-(4-fluorobenzyl) phenyl]-1-piperazinyl}-2-propanol dimethanesulfonate (SUN N8075) was synthesized at Asubio Pharma Co. Ltd. (Osaka, Japan).

### Cell cultures

Cultures of human neuroblastoma (SH-SY5Y) cells were maintained in Dulbecco's modified Eagles's medium (D-MEM, St. Louis, MO) containing 10% FBS (Valeant, Costa Mesa, CA), 100 U/ml penicillin (Meiji Seika Kaisha Ltd., Tokyo, Japan), and 100 µg/ml streptomycin (Meiji Seika Kaisha Ltd.) in a humidified atmosphere of 95% air and 5% CO_2_ at 37°C. The cells were passaged by trypsinization every 5 to 7 days, as described in a previous report [14].

### ER stress-induced cell death

To examine the effects of the SUN N8075 on tunicamycin-induced cell death, cells were seeded at a density of 1×10^4^ cells per well into 96-well plates, and then incubated in a humidified atmosphere of 95% air and 5% CO_2_ at 37°C for 2 days. Tunicamycin (Wako, Osaka, Japan) or thapsigargin was added to the cells at a final concentration of 2 µg/ml and 2 µM, respectively. SUN N8075 was added 1 h before the tunicamycin treatment, at which time the cell-culture medium was replaced with D-MEM containing 1% FBS. Assessment of cell viability was performed using two methods, each at 24 h after the addition of tunicamycin. The first method was a single-cell digital imaging-based method employing fluorescent staining of nuclei. Cell death was assessed on the basis of combination staining with fluorescent dyes [namely, Hoechst 33342 and YO-PRO-1 (Molecular Probes, Eugene, OR)], observations being made using an OLYMPUS IX70 inverted epifluorescence microscope (OLYMPUS, Tokyo, Japan). Hoechst 33342 freely enters living cells and therefore stains the nuclei of viable cells, as well as those that have suffered apoptosis or necrosis. Apoptotic cells can be distinguished from viable and necrotic cells on the basis of nuclear condensation and fragmentation. YO-PRO-1 is a membrane-impermeant dye that is generally excluded from viable cells, whereas early-stage apoptotic and necrotic cells are YO-PRO-1-positive. At the end of the culture period, Hoechst 33342 and YO-PRO-1 dyes were added to the culture medium (at 8 and 0.1 µM, respectively) for 30 min. Images were collected using a digital camera (COOLPIX 4500, Nikon, Tokyo, Japan). In a blind manner, a total of at least 400 cells per condition were counted using image-processing software (Image-J ver. 1.33f; National Institutes of Health, USA). As the second method for measuring cell viability, cell metabolic activity was quantitatively assessed. Cell viability was assessed following immersion in 10% WST-8 solution [2-(2-methoxy-4-nitrophenyl)-3-(4-nitrophenyl)-5-(2,4-disulfophenyl)-2H-tetrazolium, monosodium salt] with an electron carrier, 1-Methoxy PMS (1-Methoxy-5-methylphenazinium, methyslsulfate) (Cell Counting Kit-8, Dojin Kagaku, Kumamoto, Japan) for 3 h at 37°C, and absorbance was recorded at 450 nm [15]. WST-8 is bioreduced by cellular dehydrogenases to an orange formazan product that is soluble in cell culture medium. The amount of formazan produced is directly proportional to the number of living cells. This absorbance is expressed as a percentage of that in control cells, after subtraction of background absorbance.

### Immunoblotting

SH-SY5Y cells or mouse spinal cords were lysed using a cell-lysis buffer [RIPA buffer (R0278; Sigma) with protease (P8340; Sigma) and phosphatase inhibitor cocktails (P2850 and P5726; Sigma), and 1 mM EDTA]. Lysates were solubilized in SDS-sample buffer, separated on 10% SDS-polyacrylamide gels, and transferred to PVDF membrane (Immobilon-P; Millipore, Bedford, MA). Transfers were blocked for 1 h at room temperature with 5% Blocking One-P (Nakarai Tesque, Inc., Kyoto, Japan) in 10 mM Tris-buffered saline with 0.05% Tween 20 (TBS-T), then incubated overnight at 4°C with the primary antibody. The transfers were then rinsed with TBS-T and incubated for 1 h at room temperature in horseradish peroxidase goat anti-rabbit or goat anti-mouse (Pierce, Rockford, IL) diluted 1∶2000. The immunoblots were developed using chemiluminescence (Super Signal® West Femto Maximum Sensitivity Substrate; Pierce), and visualized with the aid of a digital imaging system (FAS-1000; TOYOBO CO., LTD, Osaka, Japan). The primary antibodies used were as follows: mouse anti-cleaved caspase-3 (#9661, Cell Signaling, Beverly, MA), rabbit anti-VGF (SantaCruz, Santa Cruz, CA) and rabbit anti-β-actin (clone AC-74, Sigma-Aldrich, Saint Luis, MO).

### Real-time PCR

To examine the effect of SUN N8075 on VGF mRNA expression, SH-SY5Y cells were seeded in 96-well plates at a density of 1.0×10^4^ cells per well. After the cells had been incubating for 48 h, they were exposed to tunicamycin at 2 µg/ml with or without SUN N8075 at 3 µM in 1% FBS DMEM for 2, 6, or 12 h. Quantitative real-time PCR was performed using a Thermal Cecler Real Time System (TP-800, Takara) with a TaqMan® Gene Expression Cells-to-CT™ Kit (Applied Biosystems) according to the manufacturer's protocol. mRNA expression was measured by real-time PCR using TaqMan probe (TaqMan Gene Expression Assay): VGF (Assay ID Details: Hs00705044_s1). The thermal cycler conditions were as follows: 2 min at 50°C and then 10 min at 95°C, followed by two-step PCR for 50 cycles consisting of 95°C for 15 sec followed by 60°C for 1 min. For each PCR, we obtained the slope value, R2 value, and linear range of a standard curve of serial dilutions. The results are expressed relative to the GAPDH (#4333764T, Applied Biosystems) internal control.

### VGF knockdown and overexpression

For VGF knockdown, the following siRNA sequences specific to human VGF were used: 5′-AUUCACUCGGGUCAGCGUGUGCGUG-3′ (sense), 5′-CACGCACACGCUGACCCGAGUGAAU-3′ (antisense). Stealth™ RNAi Negative Control Medium GC Duplex #2 was used as a control. All siRNAs were obtained from Invitrogen (Carlsbad, CA, USA). The SH-SY5Y cells were transfected with 50 pg of siRNA using Lipofectamine™ RNAi MAX Reagent (Invitrogen) according to the manufacturer's protocol.

For overexpression of VGF, pCMV6-ENTRY containing myc-tagged VGF and same empty vector (control) were obtained from ORIGENE (Rockville, MD, USA). SH-SY5Y cells were transiently transfected with each plasmid using Lipofectamine™2000 (Invitrogen) according to the manufacturer's protocol. The transfected cells were subjected to immunoblot analysis and cell death assay. The cells transiently transfected with plasmids or siRNAs were incubated for 24 h at 37°C in a humidified atmosphere of 95% air and 5% CO_2_.

### Animals

We used two types of transgenic animals overexpressing mutated human SOD1 genes: transgenic mice overexpressing mutated (glycine to alanine in position 93) human SOD1 (G93A) [16] and transgenic rats overexpressing mutated (histidine to arginine in position 46) human SOD1 (H46R) [17]. The transgenic G93A [B6SJL-Tg (SOD1-G93A) 1Gur/J] mice were purchased from the Jackson Laboratory (Bar Harbor, ME, USA). The hemizygous SOD1G93A mice were maintained by mating transgenic males with wild-type (WT) females. Mouse genotypes were determined by polymerase chain reaction analysis as previously reports [16], [18]. The transgenic male H46R rats were used a colony generated in Tohoku University Graduate School of Medicine as a previous report [17]. All experiments were approved and monitored by the Institutional Animal Care and Use Committee of Gifu Pharmaceutical University (Permit Number: 08-085, 2008-366).

### Drugs

SUN N8075 was dissolved in 6% captisol solution, and subcutaneously administered at doses of 30 mg/kg and 10 mg/kg, respectively, once daily from 10-weeks-old (for SOD1G93A mice) or 15-weeks-old (for SOD1H46R rat) to lifetime. In control group, vehicle (6% captisol solution) was subcutaneously administered at 10 ml/kg.

### Rotarod test

A rotarod test was performed with a rotation at 5 rpm once every 7 days, as previously described by Chiba et al. [19]. Mice, which did not learn to remain on the rod for 600 s, were excluded from motor performance analysis. The disease onset was defined as the day when a mouse first dropped off the rotarod within 600 s.

### Motor score

Symptomatic disease onset and post-symptomatic disease progression were assessed using a 5-point motor score system as described in previous reports [20]. This assay was performed once per day until the disease end-point. Rats were allowed to move freely in open field and were scored blindly by a single observer (H.T.). The scoring system was used following these scales: 5, normal movement; 4, limping or dragging of any limb, however still able to stand on hindlimbs; 3, dragging of lower body and inability to stand on hindlimbs; 2, Unable to drag the lower body, however righting reflexes present from both side; 1, a righting reflex from only one side; 0, absent righting reflexes from both sides within 30 second. This point was defined as the end-point and lifespan was determined by the age of the rats at end-point. Symptomatic onset was identified when the score showed 4.

### Survivals

Mortality was scored at the day when the mouse was unable to right itself within 30 s after being placed on its back.

### Microarray analysis

Total RNA was extracted from SH-SY5Y using the RNeasy Mini kit (Qiagen, Westburg, Leusden, NL, USA) including a DNase digestion step, according to the manufacturer's instructions. Total RNA was amplified and labeled with Cyanine 3 for the test sample using Agilent's Low RNA Input Linear Amplification Kit (Agilent Technologies, Palo Alto, CA) following the detailed protocol described in the kit manual (version 5.7). Briefly, 500 ng of total RNA was reversed transcribed to double-strand cDNA using a poly dT-T7 promoter primer. Primer, template RNA and quality-control transcripts of known concentration and quality were first denatured at 65°C for 10 min and incubated for 2 hours at 40°C with 5× first strand Buffer, 0.1 M DTT, 10 mM dNTP, MMLV RT, and RNase-out. The MMLV-RT enzyme was inactivated at 70°C for 15 min. cDNA products were then used as templates for in vitro transcription to generate fluorescent cRNA. cDNA products were mixed with a transcription master mix in the presence of T7 RNA polymerase and Cy3 labeled-CTP and incubated at 40°C for 2 h. Labeled cRNAs were purified using Qiagen's RNeasy mini spin columns and eluted in 30 µL of nuclease-free water. After amplification and labeling, cRNA quantity and cyanine incorporation were determined using a NanoDrop ND. Microarray expression experiments were performed on 4×44 K Agilent Human and Mouse expression arrays (Agilent technologies) by hybridizing SH-SY5Y cells according to the manufacturer's instructions. Images of the arrays were acquired using a microarray scanner G2565BA (Agilent technologies) and image analysis was performed using Feature Extraction software version 9.5 (Agilent Technologies).

### Gene expression data analyses (statistical analysis and hierarchical clustering analysis)

Raw data was imported into GeneSpring GX 7.3.1 (Agilent Technologies) and normalized by setting all measurements <0.01 to 0.01, normalizing each chip to the 75th percentile of all measurements taken for that chip, and normalizing each gene to the median measurement for that gene across all chips. To focus on genes with reliable measurements, the normalized data were filtered for data that had present call in ≥33% of all samples (based on flag information of Agilent Feature Extraction Software v.9.5.3) and the genes chosen by parametric test (don't assume variances equal) with a Bejamini and Hochberg false discovery rate (FDR) <0.01 and twofold restriction filters were utilized. Hierarchical clustering was performed on the log transformed normalized data of all the samples. Pearson's correlation was used for the similarity metric and the calculation result was visualized using “Gene Tree” algorism. A red/blue color scheme was used in the heat maps of the gene expressions.

### Tissue Preparation

SOD1G93A and WT mice at 14 weeks of age (decreasing motor function) were anesthetized with sodium pentobarbital (80 mg/kg, i.p.) (Nembutal, Dainippon, Osaka, Japan) and perfused with 2% (w/v) paraformaldehyde solution in 0.01 M phosphate-buffered saline (PBS) at pH 7.4. Spinal cord tissues were removed after a 15-min perfusion at 4°C and immersed in the same fixative solution for 24 h. Each spinal cord included L1, L2, and L3 levels, were soaked in 25% (w/v) sucrose at 4°C for 1 day, and then frozen in embedding compound (Tissue-Tek, Sakura Finetechnical Co. Ltd., Tokyo, Japan). Embedded tissues were immediately frozen with liquid nitrogen and stored at −80°C. Serial transverse sections were cut on a cryostat to a thickness of 20 µm at 2-mm intervals (3 sections total for each segment) and used for cresyl violet staining or immunohistochemistry.

### Human spinal cord

Spinal cord segments were taken at autopsy from ten patients with features typical of sporadic ALS. Spinal cord tissue from 6 individuals without history/evidence of neurological or psychiatric disease was taken for the control samples (Table S1). Three paraffin-embedded coronal sections cut at 4-µm thickness through the spinal cord [cervical spinal cord (C7), thoracic spinal cord (T8), and lumbar spinal cord (L4)] were prepared in the standard manner. The research protocol was approved by the institutional ethics committee of Niigata University, and informed consent was obtained from all participants.

### Immunohistochemistry

The sections were stained with the following antibodies: (i) mouse anti-VGF monoclonal antibody (1∶50; Santa Cruz Biotechnology, Santa Cruz, CA, USA); (ii) goat anti-NeuN polyclonal antibody (1∶5000; Millipore, Bedford, MA, USA); (iii) mouse anti-GFAP monoclonal antibody (1∶1000; Millipore); and (iv) mouse anti-CD11b monoclonal antibody (1∶1000; BMA Biomedicals, Augst, Switzerland) within Can Get Signal immunostain solution A (Toyobo CO., LTD., Osaka, Japan). Sections were treated with 0.3% H_2_O_2_ in methanol for 30 min at room temperature and blocking with mouse-on-mouse blocking reagent for 1 h at room temperature. Anti-VGF antibody was applied to the sections for overnight at 4°C. After washing the sections with 0.01 M PBS, sections were incubated with biotinylated anti-mouse IgG for 2 h followed by washing, and were incubated with the avidin-biotin-peroxidase complex for 30 min at room temperature. The sections were finally visualized using diamino benzidine/H_2_O_2_ substrate for peroxidase (Vector Laboratories, Inc., Burlingame, CA).

For paraffin embedding human spinal cord sections, conventional antigen retrieval method for each antigen was performed at its optimal condition with 0.01 M sodium citrate buffer, pH 7.4. Next, coronal sections of spinal cord were washed with 0.01 M PBS, and then treated with 0.3% hydrogen peroxidase in 0.01 M PBS for 30 min at room temperature. Furthermore, the sections were preincubated with 10% normal horse serum (Vector) in 0.01 M PBS for 30 min and then incubated for 1 h at room temperature with specific goat anti-VGF polyclonal antibody (R-15; 1∶50, Santa Cruz Biotechnology, CA, USA) in the following solution: 10% normal horse serum in 0.01 M PBS containing 0.3% (v/v) Triton X-100. They were washed with 0.01 M PBS and then incubated with biotinylated anti-goat IgG before being incubated with the avidin-biotin-peroxidase complex for 30 min at room temperature, and finally visualized using diamino benzidine/H_2_O_2_ substrate. To assess intensity of VGF immunoreactivity in the Rexed laminae IX anterior horn (AH) in the human spinal cord, sections immunostained with VGF were used for the intensity measurement: C7, T8, and L4 in the spinal cords of the control patients and patients with sporadic ALS, respectively. The volume used for the intensity measurement was 438×330×4 µm (section thickness) of each side in the spinal cord. The density measurements were carried out under objective ×40 with bright-field microscope in a masked fashion by a single observer (Y.I.). Anti-VGF antibody revealed the expression pattern of VGF in spinal cord. The density on each section was measured using MetaMorph software (Molecular Devices). Data from each section was averaged for each spinal cord, and the values obtained were used to evaluate the density of VGF immunoreactivity.

When double-immunostaining for VGF/GFAP, VGF/NeuN, or VGF/CD11b were performed, these antibodies were applied to the sections for overnight at 4°C after blocking with 0.01 M PBS containing 10% normal goat serum (Vector) or mouse-on-mouse blocking reagent (M.O.M. immunodetection kit, Vector) for 1 h. After washing the sections with 0.01 M PBS, immunoreactivity was visualized by incubating them for 2 h at room temperature with secondary antibodies conjugated with Alexa 488 rabbit anti-mouse, Alexa 546 rabbit anti-goat, Alexa 488 goat anti-rabbit, or Alexa 546 goat anti-mouse (1∶1000; Invitrogen Japan K.K., Tokyo, Japan). At the end of immunostaining, Hoechst 33342 (1∶1000) was added to the samples for 30 min to visualize nucleus.

Total images of the spinal cord were taken using a microscope (BX50; Olympus, Tokyo, Japan) fitted with ×20 and ×40 microscope objective lenses. The images visualized by diamino benzidine were taken using a charge-coupled device camera (MicroPublisher 5.0RTV, QIMAGING, Burnaby, BC, Canada) and immunofluorescence images were taken using a cooled charge-coupled device camera (DP30BP; Olympus) at 1360×1024 pixels via Metamorph (Universal Imaging Corp., Downingtown, PA, USA).

### Data Analysis

Data are presented as means ± S.E.M. Statistical comparisons were made by Dunnet's test, Tukey test, or Student's *t*-test using a SPSS 16.0 (SPSS Inc., IL, USA). Statistical analysis of the cumulative probability of survival was performed with the Kaplan–Meier life test. Statistical analysis of the mean age of onset and survival was performed with log-rank test. A value of *P*<0.05 was considered to indicate a statistical significance.

Further Materials and Methods used in the supplemental Figures and Tables were described in the supporting information (Methods S1).

## Results

### SUN N8075 protects against cell death induced by ER stress

Representative photographs in cell morphology occurring 24 h after tunicamycin treatment at 2 µg/ml are shown in Fig. 1B. An increase in non-adherent cells was observed at 24 h after tunicamycin treatment compared with that in non-treated control cells. Pretreatment with SUN N8075 at 10 µM reduced the increase in non-adherent cells. SUN N8075 at 0.3 to 10 µM concentration-dependently inhibited the reduction of cell viability 24 h after tunicamycin treatment, its effect being significant at 1, 3 and 10 µM (Fig. 1C). Furthermore, SUN N8075 at 0.3 to 3 µM concentration-dependently inhibited the reduction of cell viability 24 h after thapsigargin treatment, and its effect being significant at 1 and 3 µM (Fig. 1D). Next, we evaluated cell death using fluorescence stainings of nuclei with Hoechst 33342 and YO-PRO-1 dyes (Fig. 1E). Non-treated control cells displayed normal nuclear morphology and negative staining with YO-PRO-1 dye, which stains early apoptotic and later-stage cells (Fig. 1E). Treatment with tunicamycin for 24 h led to shrinkage and condensation of nuclei, and to positive staining with YO-PRO-1 dye (Fig. 1E). Treatment with SUN N8075 at 3 µM reduced the tunicamycin-induced morphological changes in the nuclei and the number of cells stained with YO-PRO-1 (Fig. 1E). The number of cells exhibiting YO-PRO-1 fluorescence was counted, and the positive cells were expressed as the percentage of YO-PRO-1- to Hoechst 33342-positive cells (Fig. 1E). Twenty-four hours after the treatment with tunicamycin, the percentage of YO-PRO-1-positive cells was 47.1±3.5% (*n* = 8), while in the control group, the percentage (supplemented with 1% FBS) was 10.7±1.3% (*n* = 8). Treatment with SUN N8075 at 3 µM significantly reduced the increase in YO-PRO-1-positive cells induced by tunicamycin. Exposure to tunicamycin elevated the level of caspase-3 activity, indicative of an early stage of ER stress-induced apoptosis [21]. To determine the mechanism underlying the action of tunicamycin, we examined the effect of SUN N8075 on the increases in cleaved caspase-3 induced by treatment with tunicamycin. Tunicamycin increased cleaved caspase-3 proteins (Fig. 1F), and pretreatment with SUN N8075 at 10 µM significantly reduced the increase in cleaved caspase-3 protein. On the other hand, treatment with SUN N8075 alone at 10 µM had little effect on the production of cleaved caspase-3 (versus the vehicle-treated controls). SUN N8075 has a potent antioxidant property [9], and therefore, the protective effects of SUN N8075 on ER stress-induced cell death may be derived from its antioxidative effect. However, antioxidants, *N*-acetyl-cysteine (NAC) at 1 mM or edaravone at 1 to 10 µM, did not show any effects on the reduction of cell viability after tunicamycin (Fig. S1A,B). Furthermore, NAC at 1 mM, edaravone at 10 µM or trolox at 100 µM also did not inhibit the reduction of cell viability after treatment with thapsigargin (Fig. S1C). These data suggest that the protective effects of SUN N8075 on ER stress-induced cell death are mediated by a mechanism other than antioxidant effect. On the other hand, SUN N8075 and antioxidants both inhibited the reduction of cell viability in mouse neuronal precursor cells (RGC-5) after serum deprivation or intrinsic oxidative stress induced by l-buthionine-(S,R)-sulfoximine (BSO) plus glutamate, suggesting that SUN N8075 protected cell damage against other stresses depending on the antioxidative effect (Fig. S2).

### Gene expression analyses

ER stress activates signaling pathways, including the unfolded protein response (UPR) that counteracts the effects of the original stress. BiP acts as an ER-resident molecular chaperone that is induced by ER stress, and this protein refolds the unfolded proteins, thereby tending to maintain homeostasis in the ER [22], [23]. C/EBP-homologous protein (CHO) is a member of the CCAAT/enhancer-binding protein family that is induced by ER stress and participates in ER-mediated apoptosis [24]. UPR is mediated by three types of ER transmembrane proteins: inositol-requiring protein-1 (IRE1), RNA-dependent protein kinase–like ER eukaryotic translation initiation factor 2α kinase (PERK), and activating transcription factor 6 (ATF6) [1], and expression of both BiP and CHOP mRNAs are upregulated by activation of these pathways. To determine whether SUN N8075 can affect the UPR after ER stress, BiP and CHOP mRNAs was quantitated with real-time PCR. Tunicamycin induced BiP and CHOP expression in a time-dependent manner (Fig. S3). SUN N8075 at a concentration of 3 µM did not show any effects on these increases after treatment with tunicamycin (Fig. S3). Furthermore, we examined cell viability and protein expression of ATF4, BiP and CHOP after tunicamycin with or without SUN N8075 in RGC-5 (Fig. S4)). SUN N8075 inhibited the reduction of cell viability induced by tunicamycin (Fig. S4A), but it did not affect the expression of ATF4, BiP, or CHOP protein at 24 h after tunicamycin treatment (Fig. S4B). These data suggest that SUN N8077 does not affect the UPRs themselves.

**Figure 2:**
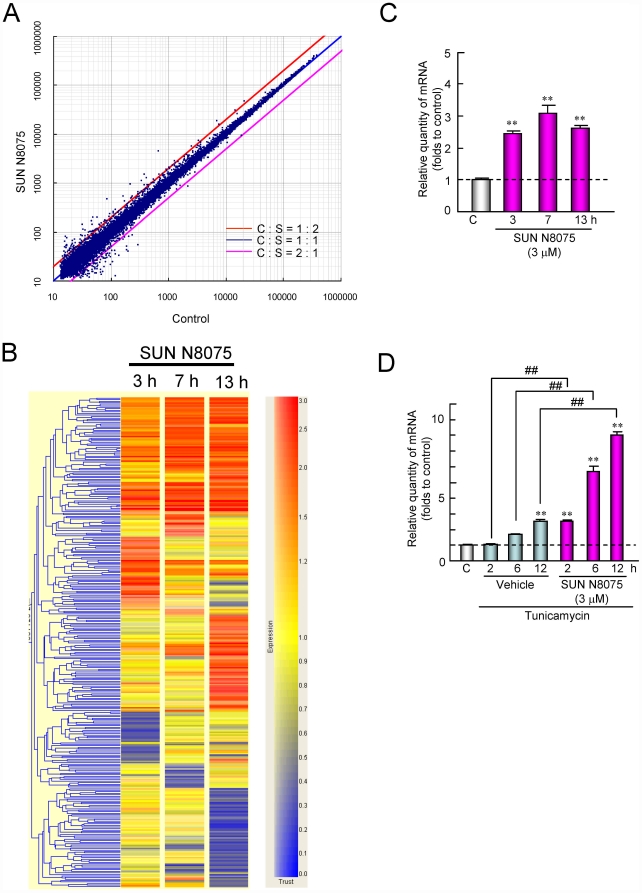
Changes in the gene expression of SH-SY5Y cells after SUN N8075 treatment. (A) Representative scatter plot comparison in gene expression with DNA microarray between SUN N8075 and vehicle control treatments at 7 h. (B) Heat map representation of hierarchical clustering of 320 genes with at least 2-fold changed expression in SH-SY5Y cells treated with 3 µM of SUN N8075. The columns and rows represent samples from the time-course study and individual gene expression levels, respectively. Shades of red indicate elevated expression while shades of blue indicate decreased expression relative to the median (see the color scale). (C, D) Quantitative analysis of *VGF* mRNA after SUN N8075 (C), and after treatment with tunicamycin with or without SUN N8075 (D).

To generate gene expression profiles after treatment with SUN N8075 or tunicamycin, we performed microarray analyses on 4×44 K Agilent Human expression arrays by hybridizing RNA from SH-SY5Y. At least more than 26,000 genes out of 41,000 gene probes on the array were detected in each sample, and a representative scatter plot comparison in gene expression with DNA microarray between SUN N8075 and vehicle control treatments at 7 h is shown in Fig. 2A. Heat map representation of the hierarchical clustering of 320 genes with at least 2-fold changed expression in SH-SY5Y cells treated with SUN N8075 at 3 µM for 3, 7 and 13 h out of the top 10% of fluorescence intensity in the non-treated control is shown in Fig. 2B and listed in Table S2. In the 320 genes, we identified the *VGF* gene as a candidate gene. The induction of *VGF* was also confirmed by real-time RT-PCR (Fig. 2C,D). SUN N8075 at a concentration of 3 µM significantly increased the *VGF* mRNA level to more than 2-fold during 13 h compared with the vehicle-treated control (Fig. 2C). Furthermore, *VGF* mRNA levels were time-dependently increased by tunicamycin, and the increase was significant for 12 h (Fig. 2D). The tunicamycin-induced *VGF* mRNA upregulation was potentiated by pretreatment with SUN N8075 at 3 µM (Fig. 2D). VGF is a neurotrophin-inducible and activity-regulated gene product expressing in the central and peripheral neurons, in which it is processed into peptides and secreted [11], [25]. VGF synthesis is stimulated by brain-derived neurotrophic factor (BDNF), a critical regulator of hippocampal development and function [26], [27]. However, the expression of neurotrophins, including *BDNF* and glial cell derived neurotrophic factor (*GDNF*), and neuropeptide Y (*NPY*) was not affected by SUN N8075 alone or after tunicamycin with or without SUN N8075 (Table 1). These results indicate that SUN N8075-induced VGF expression is not mediated by neurotrophins. On the other hand, SUN N8075 increased the expression of the immediate early genes (IEGs), *c-fos* and early growth response gene 1 (*EGR1*) but not cAMP response element-binding protein 1 (*CREB1*) or *JUN* (Table 1). IEGs are regulatory transcription factors, which may promote VGF expression. Alder et al. [11] reported that BDNF increases the expression of IEGs, including *c-fos* and *EGR1* at 20 min and *VGF* at 3 h, and they are blocked by mitogen-activated protein kinase kinase (MEK) inhibitor or Ca^2+^-calmodulin-dependent protein kinase II (CaMKII) inhibitor. These findings suggest that SUN N8075 may induce VGF by regulating mitogen-activated protein kinase (MAPK) or CaMKII activity. On the other hand, almost all the 15 ER-related genes listed in Table S3 were increased after being treated with tunicamycin, but SUN N8075 did not affect the expression of ER-related genes, suggesting that SUN N8075 could not act on the UPRs themselves.

**Table 1:** Gene expression of *VGF*, neurotrophins and gene transcription factors in SH-SY5Y cells after SUN N8075 and during ER stress with or without SUN N8075.

		Fold change (vs. control)						
					6 h after Tm		12 h after Tm	
Gene	Genbank	Time after SUN N8075			Vehicle	SUN	Vehicle	SUN
Symbol	Accession	3 h	7 h	13 h	7 h	7 h	13 h	13 h
*VGF*	NM_003378	1.25	1.94	2.21	1.12	2.72	2.43	6.78
*NPY*	NM_000905	1.22	1.43	1.64	0.57	0.84	0.82	1.20
*BDNF*	NM_170735	0.82	0.85	0.94	0.86	0.69	0.58	0.58
*GDNF*	AJ001898	1.12	1.04	0.93	0.91	0.84	0.91	0.89
*FOS*	NM_005252	2.42	1.67	1.76	1.01	3.29	1.14	1.86
*EGR1*	NM_001964	2.75	1.93	1.73	2.61	11.19	1.15	2.63
*JUN*	NM_002228	0.72	1.00	0.94	2.63	2.26	2.02	1.86
*CREB1*	NM_134442	1.08	1.09	1.02	1.04	1.21	0.87	0.95

SUN N8075 at a concentration of 3 µM was treated for 3, 7 or 13 h. One hour before tunicamycin treatment, SUN N8075 was treated, then tunicamycin was treated for 6 (7 h after SUN) or 12 h (13 h after SUN). *VGF*: VGF nerve growth factor inducible, *NPY*: Neuropeptide Y, *BDNF*: Brain-derived neurotrophic factor, *GDNF*: Glial cell derived neurotrophic factor, FOS: c-fos proto-oncogene, *EGR1*: Early growth response protein 1, *JUN*: Jun oncogene, *CREB1*: cAMP responsive element binding protein 1, SUN: SUN N8075, Tm: tunicamycin.

### Requirement of VGF expression for the protective effects of SUN N8075 on ER stress-induced cell death

**Figure 3:**
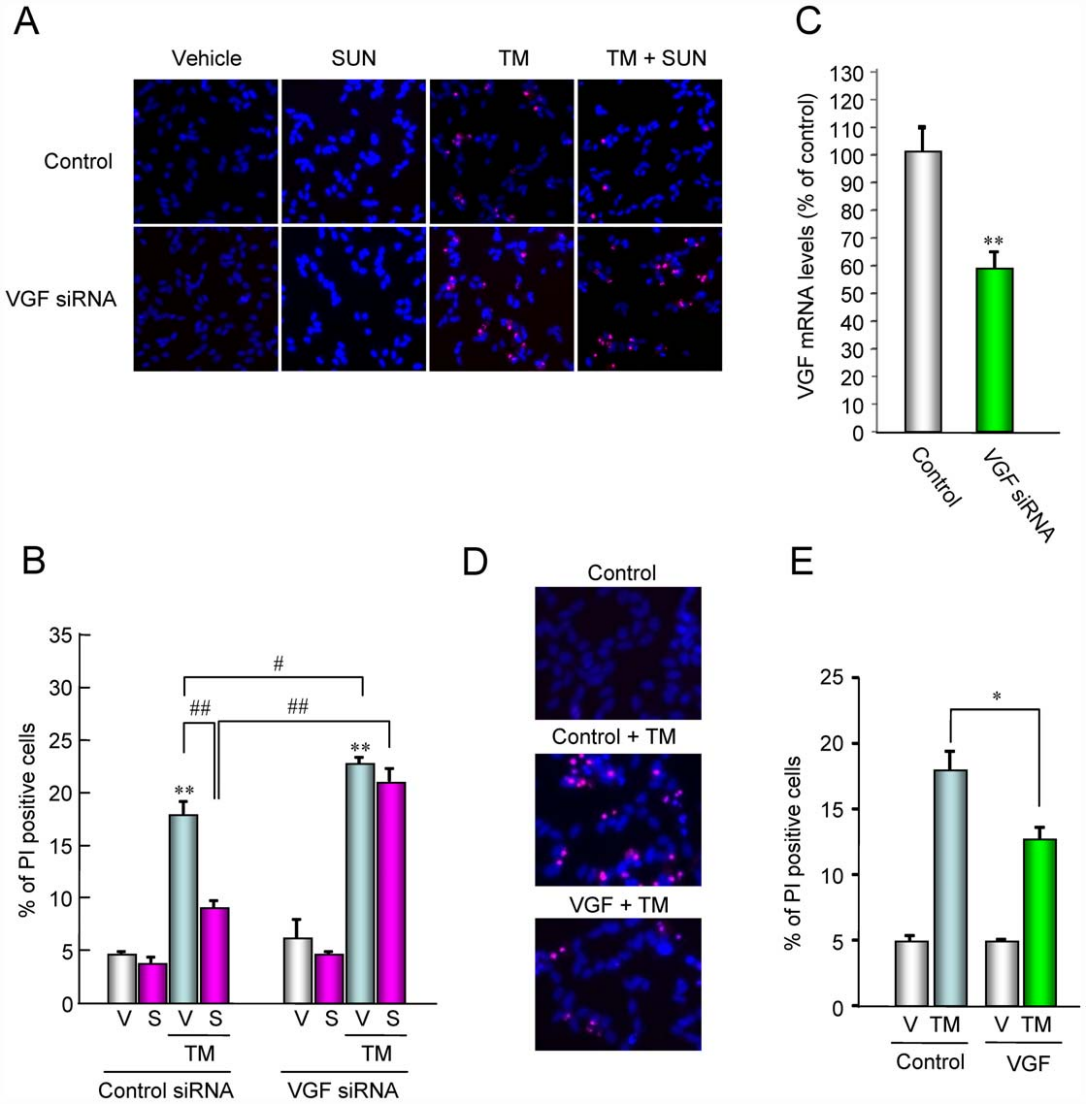
VGF protects cells against ER stress-induced SH-SY5Y cell death. (A) Representative fluorescence microscopy showing nuclear stainings for Hoechst 33342 (blue) and propidium iodide (green) on SHSY-5Y cells transfected VGF siRNA or control siRNA at 24 h after tunicamycin (TM) treatment with or without SUN N8075 (SUN). (B) The number of cells displaying fluorescence was counted, and positive cells were expressed as the percentage of propidium iodide to Hoechst 33342. Each column represents the mean ± S.E.M. (*n* = 6). #*p*<0.05, ** and ##*p*<0.01 versus relevant control group. (C) VGF siRNA transfection reduced the VGF mRNA level in SH-SY5Y cells. Each column represents the mean ± S.E.M. (*n* = 6). **p<0.01 versus control group. (D, E) VGF overexpression inhibited cell deathinduced by tunicamycin. *p<0.05 versus TM plus empty vector-transfected control control group.

We tested the involvement of VGF in the protective effect of SUN N8075 on ER-induced cell death in SH-SY5Y cells. As shown in Fig. 3A and 3B, the *VGF* siRNA completely abolished the protective effect of SUN N8075 against tunicamycin-induced cell death. Furthermore, *VGF* siRNA aggravated tunicamycin-induced cell death compared with the control siRNA (Fig. 3B), suggesting that endogenous VGF protects cells under ER stress. In this condition, *VGF* siRNA decreased the *VGF* mRNA to 60% of the control (Fig. 3C). On the other hand, overexpression of VGF transiently transfected VGF plasmid vector inhibited cell death induced by tunicamycin compared with the empty vector-transfected control (Fig. 3D,E). These results strongly suggest that the protective effect of SUN N8075 on tunicamycin-induced cell death is mediated by the expression of endogenous VGF. Furthermore, this is the first report to demonstrate that VGF overexpression protects against ER stress-induced cell death.

### SUN N8075 activates cell survival signals

VGF is upregulated by BDNF through extracellular signal-regulated protein kinase (ERK)-dependent phosphorylation of the nuclear transcription factor cAMP-response element binding protein 1 (CREB1) in primary cultures of hippocampal neurons [11]. Furthermore, BDNF protects neurons against apoptotic cell death through MEK/ERK and phosphatidylinositol 3-kinase (PI3K)/Akt pathways [28]. Activations of endogenous Akt and MEK/ERK also control cell survival during ER stress by directly counteracting ER stress [29]. Here we measured the phosphorylation levels of Akt (p-Akt) and ERK1/2 (p-ERK1/2) after SUN N8075 treatment or after tunicamycin treatment with or without SUN N8075. SUN N8075 increased p-Akt levels in a time- and concentration-dependent manner (Fig. S5A,B). In contrast, tunicamycin treatment for 24 h slightly reduced both pAkt and p-ERK1/2 (Fig. S5C,D). SUN N8075 inhibited the dephosphorylations of p-Akt and p-ERK1/2 at 24 h after treatment with tunicamycin (Fig. S5C,D). ER stress activates survival signals such as PI3K/Akt and MEK/ERK pathways in parallel with the UPR. Hu et al. [29] reported that Akt and ERK are activated for up to 4 h after tunicamycin or thapsigargin stimulation, and their activation is eliminated thereafter. Furthermore, Akt or MEK1 inhibition sensitized cells to ER stress-induced cell death [29]. In the present study, a PI3K inhibitor (LY294002; 20 µM) or MEK1/2 inhibitor (U0126; 5 µM) reduced cell viability, and sensitized cells to ER stress-induced cell death (Fig. S5E,F). Furthermore, the protective effect of SUN N8075 was attenuated by the pretreatment with a PI3K inhibitor (LY294002; 20 µM) or MEK1/2 inhibitor (U0126; 5 µM) (Fig. S5E,F). These data suggest that SUN N8075 enhances survival signals such as PI3K/Akt and MEK/ERK pathways. As mentioned above, VGF may be upregulated by ERK-dependent phosphorylation of CREB1 or induction of other IEGs including *c-fos* and *EGR1* after SUN N8075.

### Motor dysfunction and survival in ALS models

To confirm the effects of SUN N8075 on motor dysfunction and the survival of the familiar ALS model, we used two types of transgenic animals overexpressing mutated human SOD1 genes: transgenic mice overexpressing mutated (glycine to alanine in position 93) human SOD1 (G93A) [16] and transgenic rats overexpressing mutated (histidine to arginine in position 46) human SOD1 (H46R) [17]. Treatment with SUN N8075 was started when the G93A mice were 10 weeks old and the H46R rats were 15 weeks old before each disease onset.

**Figure 4:**
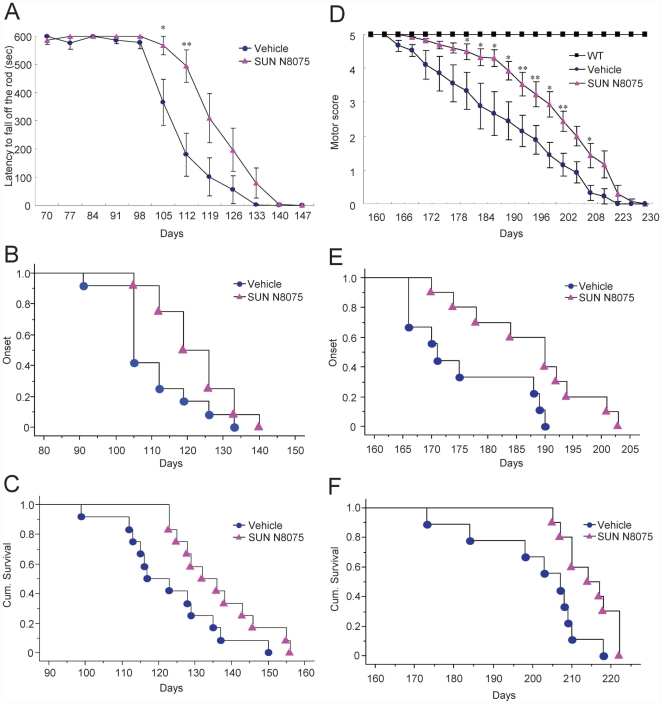
Effects of SUN N8075 on motor dysfunction, disease onset and survival in two types of familial ALS model animals. (A–C) G93A mutant SOD1 mice; effects of SUN N8075 at 30 mg/kg/day, s.c., on motor performance evaluated by the rotarod test (A), cumulative probability of the onset of motor deficit (B), and survival (C) in G93A mutant SOD1 mice. (D–F) H46R mutant SOD1 rats; effects of SUN N8075 at 10 mg/kg/day, s.c., on the motor performance score (D), cumulative probability of the onset of motor deficit (E), and survival (F) in H46R mutant SOD1 rats.

In the G93A mice, motor performance was tested by a rotarod test (Fig. 4A). Vehicle-treated G93A mice began to show a decline in motor performance around the age of 100 days that continued to be gradually impaired thereafter; the mice were unable to perform the test around the age of 130 days. Treatment with SUN N8075 (30 mg/kg/day s.c.) prolonged the decline of latency to fall off the rod. Kaplan–Meier life curves suggested that treatment with SUN N8075 prolonged disease onset (*p* = 0.024) and survival (*p* = 0.0351) in the G93A mice (Fig. 4B,C). In the G93A model mice, the mean survival days of the vehicle-treated and the SUN N8075-treated mice were 122.8±4.0 (*n* = 12) and 136.2±3.4 (*n* = 12), respectively (Fig. 4C). Treatment with SUN N8075 significantly prolonged the mean lifespan by 10.9% (*p* = 0.0178) in the G93A mice compared with that in the vehicle-treated G93A mice. On the other hand, there was no significant difference in the course of body weight between the SUN N8075-treated and the vehicle-treated G93A mice (Fig. S6A).

Next, we used SOD1 H46R mutant rats as other types of familial ALS models to evaluate the effects of SUN N8075 on motor dysfunction and survival. H46R is one of 119 known SOD1 mutations that cause familial ALS, and H46R rats are characterized by initial muscle weakness and atrophy in the legs, very long clinical courses and include many neuropil aggregates that lack vacuoles [30]. In the H46R rats, the motor score was determined by a 5-point motor score system (Fig. 4D). The vehicle-treated H46R rats began to show a decline in motor performance around the age of 170 days that continued to be gradually impaired thereafter; the mice were unable to perform the test around the age of 220 days. Treatment with SUN N8075 (10 mg/kg/day s.c.) prolonged the decline of the motor score. Kaplan–Meier life curves suggested that treatment with SUN N8075 prolonged disease onset (*p* = 0.012) and survival (*p* = 0.011) in H46 rats (Fig. 4E,F). In the H46R rats, the mean survival days of the vehicle-treated and SUN N8075-treated rats were 201.1±4.7 (*n* = 9) and 214.7±2.0 (*n* = 10), respectively (Fig. 4F, Table S4). Treatment with SUN N8075 significantly prolonged the mean lifespan by 6.7% (*p* = 0.012) in the H46R rats compared with that in the vehicle-treated H46R rats. On the other hand, there was no significant difference in the course of body weight between the SUN N8075-treated and the vehicle-treated H46R rats (Fig. S6B).

In this study, SUN N8075 was administered by subcutaneous injection at 30 mg/kg for mice and at 10 mg/kg for rats, and the maximum concentrations in plasma were approximately 2 µM and 1 µM, respectively (Tamura S. et al., unpublished data). In the present study, SUN N8075 at concentrations of 1 µM or higher inhibited ER stress-induced cell death *in vitro*. Taken together, these results show that SUN N8075 could delay disease onset and prolong survival in different two types of familial ALS models.

### Motor neuron loss and VGF expression in the spinal cords of G93A ALS mice and sporadic ALS patients

**Figure 5:**
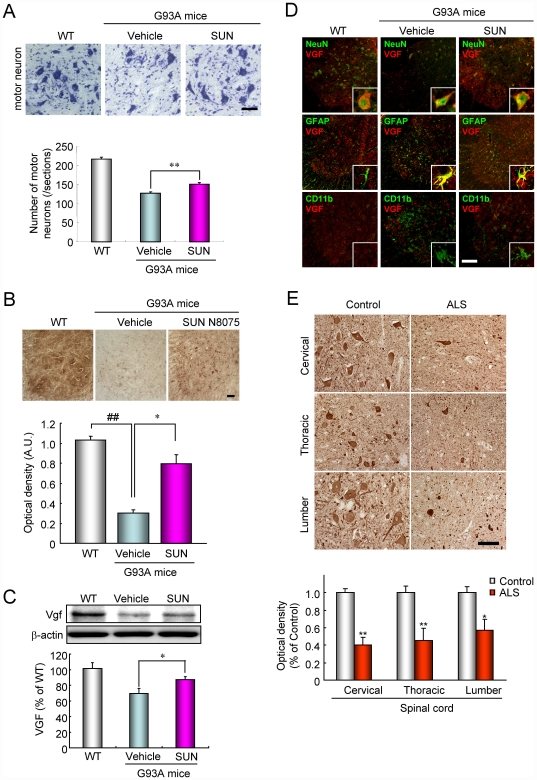
Effects of SUN N8075 on the decreased motor neurons and VGF immunoreactivity of the spinal cords of SOD1G93A mice and sporadic ALS patients. (A) cresyl violet staining, (B) VGF immunostaining, (C) VGF immunoblotting, and its double-immunostainings, (D) with GFAP (activated astrocyte marker), NeuN (neuronal marker) or GD11b (activated microglial marker). Scale bars  = 50 µm. Each column represents the mean ± S.E.M. (n = 6). *p<0.05, ** and ##*p*<0.01 versus the relevant control group. (E) Human spinal cord tissue section. Representative photographs are shown for the anterior horns of the spinal cords in the control patients and the patients with sporadic ALS. Scale bar  = 100 µm. Average density of VGF immunoreactivity in the anterior horn of the human spinal cord. Each column represents the mean ± S.E.M. (*n* = 6). *p<0.05, **p<0.01 versus control.

We evaluated the effect of SUN N8075 on motor neuron loss in the spinal cords of G93A mice at 14 weeks of age. The number of motor neurons in the lumbar anterior horn of the G93A mice was decreased to 59% of their wild-type (WT) littermates (Fig. 5A). Treatment with SUN N8075 (30 mg/kg, s.c./day) in the G93A mice significantly increased the number of surviving motor neurons compared with that in the vehicle-treated G93A mice (Fig. 5A). Recent studies revealed that a lowering VGF-derived 4.8 kDa fragment was identified in the CSF from patients with ALS [31] and that VGF content was decreased in the CSF of ALS patients and in the CSF, serum and spinal cord motor neurons of G93A mice [13]. In the present study, we used immunohistochemistry (Fig. 5B) and Western blotting (Fig. 5C) to assess the expression of the VGF protein in the spinal cord. A large number of VGF-positive cells were observed in the lumbar anterior horn of the WT littermates (Fig. 5B). VGF immunoreactivity was markedly decreased in the spinal cords of GA93A mice compared with that in their WT littermates, and the reduction was significantly ameliorated by treatment with SUN N8075. Western blot analysis also revealed that a single VGF band at approximately 90 kDa was identified, and the band intensity in the spinal cord of the G93A mice was significantly decreased to 58% of that of the WT mice (Fig. 5C). Treatment with SUN N8075 inhibited the decrease in the VGF band intensity in the spinal cords of the G93A mice (Fig. 5C). Furthermore, we used double-immunofluorescent staining to determine VGF localization in the lumbar anterior horn of the spinal cord (Fig. 5D). VGF colocalized with NeuN-positive motoneuron (>25 µm diameter) but not GFAP (an astrocyte marker)- or CD11b (an activated microglial marker)-positive glial cells in the lumbar anterior horn of the WT littermates (Fig. 5D). On the other hand, in the lumbar anterior horns of the G93A mice, VGF-immunoreactive signals colocalized with GFAP-positive astrocytes in addition to motor neurons but not the CD11b-positive glial cells (Fig. 5D). These data indicate that VGF expression is reduced in the spinal cords of the G93A mice, and the decrease is ameliorated by the treatment with SUN N8075. As mentioned above, the VGF level has been reported to be decreased in the CSF of ALS patients compared with normal subjects [13], but there is no evidence of the decrease in VGF in the spinal cords of ALS patients. In the present study, the expression level of VGF in postmortem spinal cords of sporadic ALS patients was markedly down-regulated, but typically expressed at high levels in the spinal cords of the control patients in any sections cervical 7 (C7), thoracic 8 (T8), and lumbar 4 (L4), respectively (Fig. 5E). The density of VGF immunoreactivity was significantly lower in the lumbar anterior horns of sporadic ALS patients than those in control patients with other diseases (0.40±0.09, 0.46±0.14, and 0.57±0.12 of the control in sections C7, T8, and L4, respectively, *n* = 6) (Fig. 5E). These data suggest that the decrease in VGF in the spinal cord is involved in ALS pathogenesis.

### Gene expression profiles and glial activation in the spinal cords of G93A ALS mice

**Figure 6:**
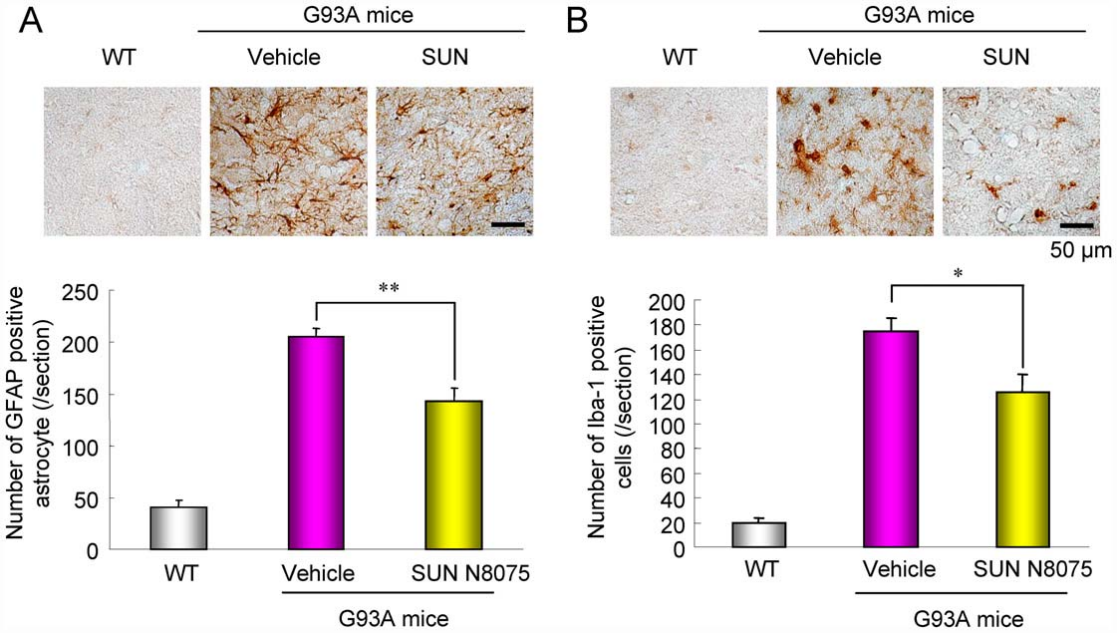
Effects of SUN N8075 on the glial activation in the spinal cords of SOD1G93A mice. G93A mice aged at 14 weeks-old increased the number of GFAP-positive astrocytes (*A*) and Iba-1-positive microglial cells (*B*) in spinal cord, and their increases were abolished by SUN N8075 treatment. Scale bars  = 50 µm. Each column represents the mean ± S.E.M. (n = 6). *p<0.05, **p<0.01 versus WT mice.

To generate gene expression profiles on the spinal cords of WT or G93A mice aged 14 weeks after SUN N8075 treatment, we performed microarray analyses on 4×44 K Agilent Mouse expression arrays. At least more than 27,000 genes out of 41,000 gene probes on the array were detected in each sample, and the Volcano plot of the probe data set averages of gene expression in the spinal cords of the G93A mice (*n* = 4) compared with the WT mice (*n* = 4) is shown in Fig. S7A. Seven hundred and twelve genes (green and red squares) were considered statistically significant between the G93A and WT mice based on the criteria of the average fold-change (FC) ≥2, p≤0.01 with the Benjamini-Hochberg correction. A heat map representation of the hierarchical clustering of the 712 different gene expressions in the spinal cords of G93A mice treated with or without SUN N8075 (each group: *n* = 4) compared with WT mice (*n* = 4) is shown in Fig. S7B. The gene expression profiles were apparent differences between SUN N8075 and vehicle treatments in the G93A mice, and a principal component analysis (PCA) component 1 discriminated significantly among the 3 groups (Fig. S7C). We analyzed the gene ontology classification for each category from the 712 different genes and listed the top 10 categories in Fig. S7D. Almost all categories included immune response genes. In particular, high intensity and variant genes (signal >1000, FC>5) were astrocyte- or microglia-related genes such as GFAP and CD11b/11c. Hence, the activation of astrocytes and microglia was determined in the spinal cords of the G93A mice using the immunohistochemical technique. The number of GFAP-positive astrocytes and Iba-1-positive microglia was significantly increased in the lumbar anterior horns of the G93A mice compared with those in their WT littermates (Fig. 6A,B). Treatment with SUN N8075 (30 mg/kg, s.c./day) significantly decreased the number of both types of glial cells in the spinal cords of the G93A mice compared with those in the vehicle-treated G93A mice (Fig. 6A,B).

## Discussion

Our results showed that SUN N8075 markedly protected against ER stress-induced cell death in SH-SY5 cells at 1 µM or higher concentrations, and the protective effects are mediated by endogenous VGF production. SUN N8075 increased VGF expression as identified by microarray and secondary validation approaches. VGF synthesis is also stimulated by BDNF through ERK-dependent phosphorylation of CREB [26], [27]. However, SUN N8075 did not enhance *BDNF* mRNA, suggesting that its *VGF* mRNA expression is not mediated indirectly by BDNF. On the other hand, SUN N8075 gradually increased the phosphorylation of ERK1/2 and Akt during the course of 24 h. These findings suggest that ERK-dependent phosphorylation of CREB1 and/or EGR1 upregulation may be crucial in regulating VGF gene expression by SUN N8075. Several types of VGF-derived peptides have been detected in the rat brain, bovine pituitary and human cerebrospinal fluid [32], [33], [34], [35], and these peptides possess multiple biological activities, autonomic activation, anti-depression, enhancement in synaptic plasticity, penile erection and increases in energy expenditure [11], [36], [37], [38]. Recently, Severini et al. [39] reported that TLQP-21, a naturally occurring VGF-derived C-terminal peptide, protects rat cerebellar granule cells against apoptosis induced by serum and K^+^ deprivation, and that TLQP-21 also increased the phosphorylation levels of ERK1/2 and Akt. Furthermore, a MEK1/2 inhibitor completely attenuated the protective effect of TLQP-21 on K^+^ deprivation-induced cell damage [39]. These findings support the idea that endogenous VGF induced by SUN N8075 may activate survival signals via the MEK/ERK and PI3K/Akt pathways.

A wide range of mechanisms are thought to be implicated in the pathogenesis of ALS, including mitochondrial dysfunction, excitotoxicity, oxidative stress, protein misfolding, proteosomal dysfunction, aberrant growth factor signaling, inflammatory process and glial activation [40], [41]. On the other hand, the accumulated evidence indicates that ER stress plays a role in the pathogeneses of familial and sporadic ALS [4], [5], [6], and therefore, they may have a common mechanism for motorneuron loss through ER stress as downstream signaling pathways. In the present study, extrinsic VGF expression protected against ER stress-induced cell death in SH-SY5Y cells. Furthermore, VGF was markedly decreased in the spinal cords of the GA93A mice compared with that in their WT littermates in agreement with a previous report [13], and the reduction was significantly ameliorated by the treatment with SUN N8075. Zhao et al. [13] also reported that exogenous VGF expression by adenoviral mouse VGF transfection in the primary spinal cord neurons from SOD1 G93A mice has been reported to protect neurons against AMPA- or NMDA-mediated excitotoxic injury. Furthermore, we demonstrated for the first time that the VGF level was lower in the spinal cords of sporadic ALS patients than in the control patients. Taken together, VGF depletion may participate in disease onset and/or progression of ALS.

In conclusion, we demonstrated that SUN N8075 inhibits ER-stress-induced neuronal cell death via VGF expression and that VGF plays a critical role in motor neuron survival. Accordingly, VGF may be a potential new therapeutic target for neurodegenerative disorders, including ALS.

## Supporting Information

Methods S1 Supplemental Materials and Methods for Supplemental Figures and Tables were addressed.
(PDF)

Figure S1 Anti‐oxidative agents do not reveal protective effects on ER stress‐induced SH‐SY5Y cell death. (*A*) Edaravone at 1 to 10 µM or (*B*) *N*‐acetyl‐cystein (NAC) at 1 mM did not show any effects on the reduction of cell viability at 24 h after tunicamycin treatment. (*C*) Edaravone (Eda) at 10 µM, NAC at 1 mM or trolox (Tro) at 100 µM did not inhibit the reduction of cell viability at 24 h after thapsigargin treatment.
(TIF)

Figure S2 SUN N8075 and anti‐oxidative agents protect cell death induced by serum devrivation, or oxidative stress in RGC‐5 cells. (*A*) SUN N8075 (SUN) at 0.1 to 1 µM, edaravone (Eda) at 10 µM, and trolox at 100 µM show protective effect on the reduction of cell viability at 48 h after serum deprivation. Each column represents the mean ± S.E.M. (n=6). ##*p*<0.01 versus control. **p<0.01 versus serum deprivation alone. (*B*) SUN N8075 (SUN) at 0.1 to 1 µM and trolox at 100 µM show protective effect on the reduction of cell viability at 24 h after l‐buthionine‐(S,R)‐sulfoximine (BSO; 0.5 mM) plus glutamate (10 mM). Each column represents the mean ± S.E.M. (n=6). ##*p*<0.01 versus control. **p<0.01 versus BSO plus glutamate alone.
(TIF)

Figure S3 SUN N8075 does not affect gene expression level of *BiP* or *CHOP* on SH‐SY5Y cells after tunicamycin. *BiP* and *CHOP* mRNA levels were measured using a quantitative real‐time PCR.
(TIF)

Figure S4 SUN N8075 protect cell death induced by tunicamycin, but did not affect protein expression of ATF4, BiP or CHOP in RGC‐5 cells. (*A*) SUN N8075 at 0.1 to 3 µM shows protective effect on the reduction of cell viability at 24 h after tunicamycin at 2 µg/ml. Each column represents the mean ± S.E.M. (n=8). ##*p*<0.01 versus control. **p<0.01 versus tunicamycin alone. (*B*) ATF4, BiP and CHOP protein levels were measured using Western blotting.
(TIF)

Figure S5 SUN N8075 enhances survival signals via Akt and ERK1/2 activations. (*A*) Time‐course of changes in phosphorylated‐Akt level after SUN N8075 treatment. (*B*) Concentration‐dependent changes in phosphorylated‐Akt level at 24 h after SUN N8075 treatment. (*C*, *D*) Tunicamycin reduced phosphorylated‐Akt (*C*) and phosphorylated‐ERK1/2 (*D*) levels, and their reductions were ameliorated by SUN N8075 treatment. (*E*, *F*) The protective effect of SUN N8075 on tunicamycin‐induced reduction of cell viability was eliminated by LY294002, a PI3kinase inhibitor, at 20 µM (*E*) or U0126, a MEK1/2 inhibitor, at 5 µM (*F*).
(TIF)

Figure S6 Effects of SUN N8075 on body weight changes in two type of familial ALS model animals. (*A*) Effect of SUN N8075 at 30 mg/kg/day, s.c. on body weight change in G93A mutant SOD1 mice. (*B*) Effect of SUN N8075 at 10 mg/kg/day, s.c. on body weight change in H46R mutant SOD1 rats.
(TIF)

Figure S7 (*A*) Volcano plot of probe data set averages of gene expressions in the spinal cords of G93A (*n*=4) compared with WT (*n*=4). The x‐axis and y‐axis correspond to the average fold‐change (FC), and the negative log10‐transformed p value between G93A and WT mice, respectively. Green (FC ≥2, 0.01≤p≤0.05) and red (FC ≥2, p≤0.01) squares represent significantly differentially expressed probe sets, and gray squares represent probe sets with no significant difference between G93A and WT mice. The changes in gene expression were considered statistically significant based on the criteria of FC ≥2, p≤0.01 with Benjamini‐Hochberg correction. (*B*) Heat map representation of hierarchical clustering of 712 different gene expressions in the spinal cords of G93A compared with WT mice. The columns and rows represent the tissue samples (WT = Blue, G93A = red, G93A + SUN N8075 = yellow) and individual gene expressions, respectively. Shades of red indicate elevated expression while shades of blue indicate decreased expression relative to the median (see the color scale). (*C*) Principal components analysis (PCA) in the spinal cord of WT, G93A and G93A + SUN N8075 treated mice. Differently expressed 712 genes in the two groups (WT vs. G93A mice) were analyzed using a principal component analysis, and the contribution rates of genes to PCA component 1, 2, and 3 were 81.23%, 6.99%, and 3.90%, respectively. The results are expressed as a one‐dimensional function with PCA component 1 that defines the direction of greatest variation in the probe/gene transcriptomic feature space. ##*p*<0.01, *p<0.05, Student's *t*‐test. (*D*) Gene ontology classification for each category from the 712 different genes.
(TIF)

Table S1 Clinical information about the spinal cord tissues from patients with sporadic ALS (a) and for control (b). M, male; F, female; PMI, post mortem interval; y, years; m, months; h, hours.
(PDF)

Table S2 Gene expression profiles after SUN N8075 treatment in SH‐SY5Y cells.
(PDF)

Table S3 Gene expression profiles for ER‐stress responsible genes after SUN N8075 alone, or tunicamycin with or without SUN N8075 in SH‐SY5Y cells. ↑: two‐fold or more increase, →: no changes.
(PDF)

Table S4 SUN N8075 prolongs disease onset and lifspan in a rat model of familial ALS. Data are the means ± S.E. (n=9 or 10), * P<0.05 vs vehicle‐treated ALS mouse group. a Observable functional deficits (motor score of 4), b Righting reflex failure (motor score of 1), c Motor score of 0.
(PDF)
