# Supplementary material for: An Inducer of VGF Protects Cells against ER Stress-Induced Cell Death and Prolongs Survival in the Mutant SOD1 Animal Models of Familial ALS
Source: PLoS One. 2010 Dec 9;5(12):e15307. doi: 10.1371/journal.pone.0015307 (PMC3000345; doi:10.1371/journal.pone.0015307)
Supplement: Methods S1 — Supplemental Materials and Methods for Supplemental Figures and Tables were addressed. (PDF) [file pone.0015307.s001.pdf]

Supplemental Materials and Methods

Chemicals

Edaravone, *N*-acetyl-cysteine, and trolox were purchased from Tocris Cookson Ltd. (Bristol, UK), Calbiochem (San Diego, CA), and Sigma-Aldrich (St. Louis), respectively. LY294002 and U0126 were from Calbiochem.

Cell cultures

Mouse neuronal precursor cells (RGC-5) were maintained in Dulbecco's modified Eagles's medium (D-MEM, St. Louis, MO) containing 10% FBS (Valeant, Costa Mesa, CA), 100 U/ml penicillin (Meiji Seika Kaisha Ltd., Tokyo, Japan), and 100 µg/ml streptomycin (Meiji Seika Kaisha Ltd.) in a humidified atmosphere of 95% air and 5% CO_2 at 37°C. The cells were passaged by trypsinization every 5 to 7 days, as described in a previous report [1].

Serum deprivation-, oxidative stress- or tunicamycin-induced cell death

To examine the effects of SUN N8075 and anti-oxidants (edaravone and trolox) on the cell death induced by serum deprivation or oxidative stress, a low density of RGC-5 (1×10^3 cells/well) was seeded in 96-well plates. To provide serum deprivation, RGC-5 cells were washed twice with D-MEM and replace a culture medium by serum-free medium with test compounds or vehicle, and cultured for a further 48 h. To provide oxidative stress or ER stress, 0.5 mM BSO plus 10 mM glutamate and tunicamycin, respectively, were added to these cultures for 24 h after pretreatment with each compound for 1 h,. Cell viability was assessed using Cell Counting Kit-8 for 3 h at 37°C as above mentioned.

Immunoblotting

SH-SY5Y or RGC-5 cells were lysed using a cell-lysis buffer [RIPA buffer (R0278; Sigma) with protease (P8340; Sigma) and phosphatase inhibitor cocktails (P2850 and P5726; Sigma), and 1 mM EDTA]. Lysates were solubilized in SDS-sample buffer, separated on 10% SDS-polyacrylamide gels, and transferred to PVDF membrane (Immobilon-P; Millipore, Bedford, MA). Transfers were blocked for 1 h at room temperature with 5% Blocking One-P (Nakarai Tesque, Inc., Kyoto, Japan) in 10 mM Tris-buffered saline with 0.05% Tween 20 (TBS-T), then incubated overnight at 4ºC with the primary antibody. The transfers were then rinsed with TBS-T and incubated for 1 h at room temperature in horseradish peroxidase goat anti-rabbit or goat anti-mouse (Pierce, Rockford, IL) diluted 1:2000. The immunoblots were developed using chemiluminescence (Super Signal® West Femto Maximum Sensitivity Substrate; Pierce), and visualized with the aid of a digital imaging system (FAS-1000; TOYOBO CO., LTD, Osaka, Japan). The primary antibodies used were as follows: rabbit anti-phospho-Akt (Ser473) mAb (193H12, Cell Signaling), rabbit anti-Akt (#9272, Cell Signaling), rabbit anti-phospho-ERK1/2 (Thr202/Tyr204) mAb (197G2, Cell Signaling, Beverly, MA), anti-ERK1/2 (#9102, Cell Signaling), mouse anti-BiP (BD Bioscience, San Jose, CA), rabbit anti-ATF4 (Santa Cruz, Santa Cruz, CA) and mouse anti-CHOP (Santa Cruz).

Real-time PCR

To examine the effect of SUN N8075 on *BiP* or *CHOP* mRNA expression, SH-SY5Y cells were seeded in 96-well plates at a density of 1.0×10^4 cells per well. After the cells had been incubating for 48 h, they were exposed to tunicamycin at 2 μg/ml with or without SUN N8075 at 3 μM in 1% FBS DMEM for 2, 6, or 12 h. Quantitative real-time PCR was performed using a

Thermal Cecler Real Time System (TP-800, Takara) with a TaqMan® Gene Expression Cells-to-CT™ Kit (Applied Biosystems) according to the manufacturer's protocol. mRNA expression was measured by real-time PCR using TaqMan probes (TaqMan Gene Expression Assay) as follows: *BiP* (Assay ID Details: Hs99999174_m1), or *CHOP* (Assay ID Details: Hs00358796_g1). The thermal cycler conditions were as follows: 2 min at 50°C and then 10 min at 95°C, followed by two-step PCR for 50 cycles consisting of 95°C for 15 sec followed by 60°C for 1 min. For each PCR, we obtained the slope value, R2 value, and linear range of a standard curve of serial dilutions. The results are expressed relative to the *GAPDH* (#4333764T, Applied Biosystems) internal control.

Microarray analysis

Total RNA was extracted from mouse spinal cords using the RNeasy Mini kit (Qiagen, Westburg, Leusden, NL, USA) including a DNase digestion step, according to the manufacturer's instructions. Total RNA was amplified and labeled with Cyanine 3 for the test sample using Agilent's Low RNA Input Linear Amplification Kit (Agilent Technologies, Palo Alto, CA) following the detailed protocol described in the kit manual (version 5.7). Briefly, 500ng of total RNA was reversed transcribed to double-strand cDNA using a poly dT-T7 promoter primer. Primer, template RNA and quality-control transcripts of known concentration and quality were first denatured at 65°C for 10 min and incubated for 2 hours at 40°C with 5× first strand Buffer, 0.1 M DTT, 10 mM dNTP, MMLV RT, and RNase-out. The MMLV-RT enzyme was inactivated at 70°C for 15 min. cDNA products were then used as templates for in vitro transcription to generate fluorescent cRNA. cDNA products were mixed with a transcription master mix in the presence of T7 RNA polymerase and Cy3 labeled-CTP and

incubated at 40°C for 2 h. Labeled cRNAs were purified using Qiagen's RNeasy mini spin columns and eluted in 30 μL of nuclease-free water. After amplification and labeling, cRNA quantity and cyanine incorporation were determined using a NanoDrop ND. Microarray expression experiments were performed on 4 × 44 K Agilent Human and Mouse expression arrays (Agilent technologies) by hybridizing mouse spinal cords according to the manufacturer's instructions. Images of the arrays were acquired using a microarray scanner G2565BA (Agilent technologies) and image analysis was performed using Feature Extraction software version 9.5 (Agilent Technologies).

Gene expression data analyses (principal components analysis)

Volcano plot was generated by plotting the negative log10-transformed p values between G93A and WT mice against the average fold-change in log2 scale. Principal components analysis (PCA) was performed by using mean-centered, log2-transformed normalized data set of significantly different gene expression in G93A compared with WT mice. The calculation result was visualized using "Principal components analysis" algorism.

Immunohistochemistry

The sections were stained with the following antibodies: (i) mouse anti-GFAP monoclonal antibody (1:1000; Millipore); and (ii) rabbit anti-Iba1 polyclonal antibody (1:800; Wako Pure Chemical Industries, Ltd., Osaka, Japan) within Can Get Signal immunostain solution A (Toyobo CO., LTD., Osaka, Japan). Sections were treated with 0.3% H_2O_2 in methanol for 30 min at room temperature and blocking with mouse-on-mouse blocking reagent for 1 h at room temperature. After washing the sections with 0.01 M PBS, sections were incubated with

biotinylated anti-mouse IgG for 2 h followed by washing, and were incubated with the avidin-biotin-peroxidase complex for 30 min at room temperature. The sections were finally visualized using diamino benzidine/H_2O_2 substrate for peroxidase (Vector Laboratories, Inc., Burlingame, CA).

References

1. Krishnamoorthy RR, Agarwal P, Prasanna G, Vopat K, Lambert W, et al. (2001) Characterization of a transformed rat retinal ganglion cell line. Mol Brain Res 86: 1-12.
