# Supplementary figures and images for: An Inducer of VGF Protects Cells against ER Stress-Induced Cell Death and Prolongs Survival in the Mutant SOD1 Animal Models of Familial ALS
Source: PLoS One. 2010 Dec 9;5(12):e15307. doi: 10.1371/journal.pone.0015307 (PMC3000345; doi:10.1371/journal.pone.0015307)

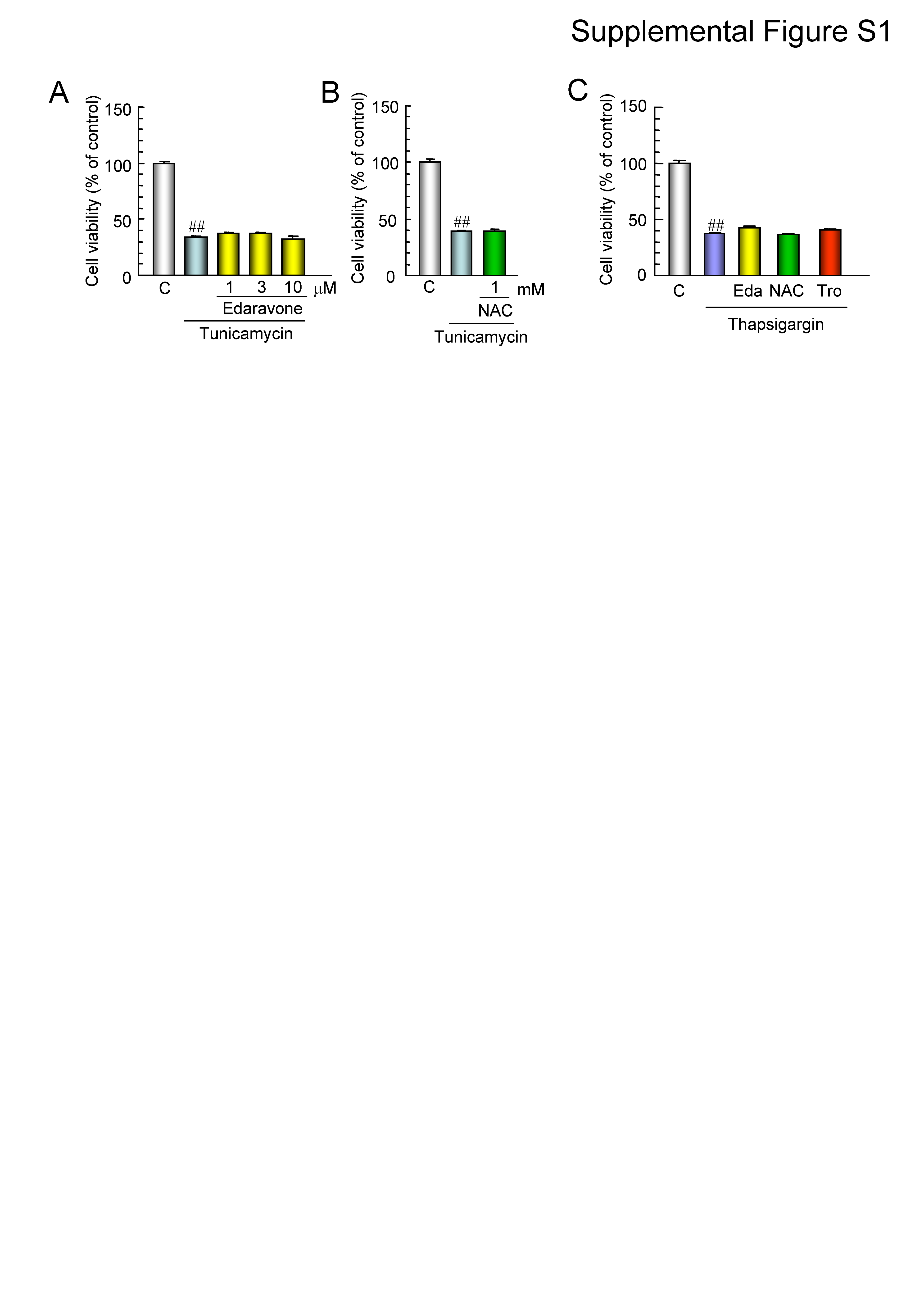

Supplement: Figure S1 — Anti‐oxidative agents do not reveal protective effects on ER stress‐induced SH‐SY5Y cell death. (A) Edaravone at 1 to 10 µM or (B) N‐acetyl‐cystein (NAC) at 1 mM did not show any effects on the reduction of cell viability at 24 h after tunicamycin treatment. (C) Edaravone (Eda) at 10 µM, NAC at 1 mM or trolox (Tro) at 100 µM did not inhibit the reduction of cell viability at 24 h after thapsigargin treatment. (TIF) [file pone.0015307.s002.tif]

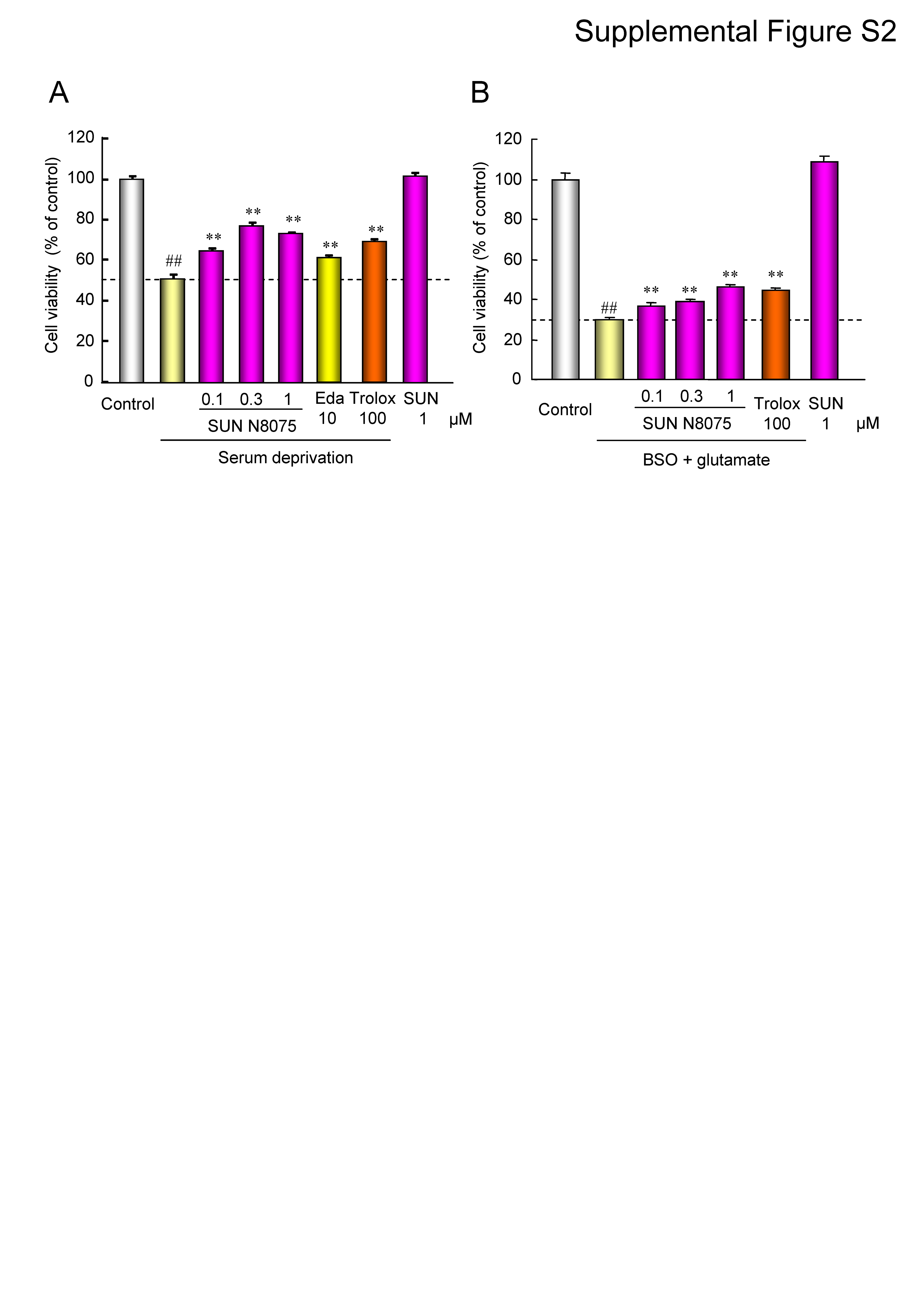

Supplement: Figure S2 — SUN N8075 and anti‐oxidative agents protect cell death induced by serum devrivation, or oxidative stress in RGC‐5 cells. (A) SUN N8075 (SUN) at 0.1 to 1 µM, edaravone (Eda) at 10 µM, and trolox at 100 µM show protective effect on the reduction of cell viability at 48 h after serum deprivation. Each column represents the mean ± S.E.M. (n=6). ##p<0.01 versus control. **p<0.01 versus serum deprivation alone. (B) SUN N8075 (SUN) at 0.1 to 1 µM and trolox at 100 µM show protective effect on the reduction of cell viability at 24 h after l‐buthionine‐(S,R)‐sulfoximine (BSO; 0.5 mM) plus glutamate (10 mM). Each column represents the mean ± S.E.M. (n=6). ##p<0.01 versus control. **p<0.01 versus BSO plus glutamate alone. (TIF) [file pone.0015307.s003.tif]

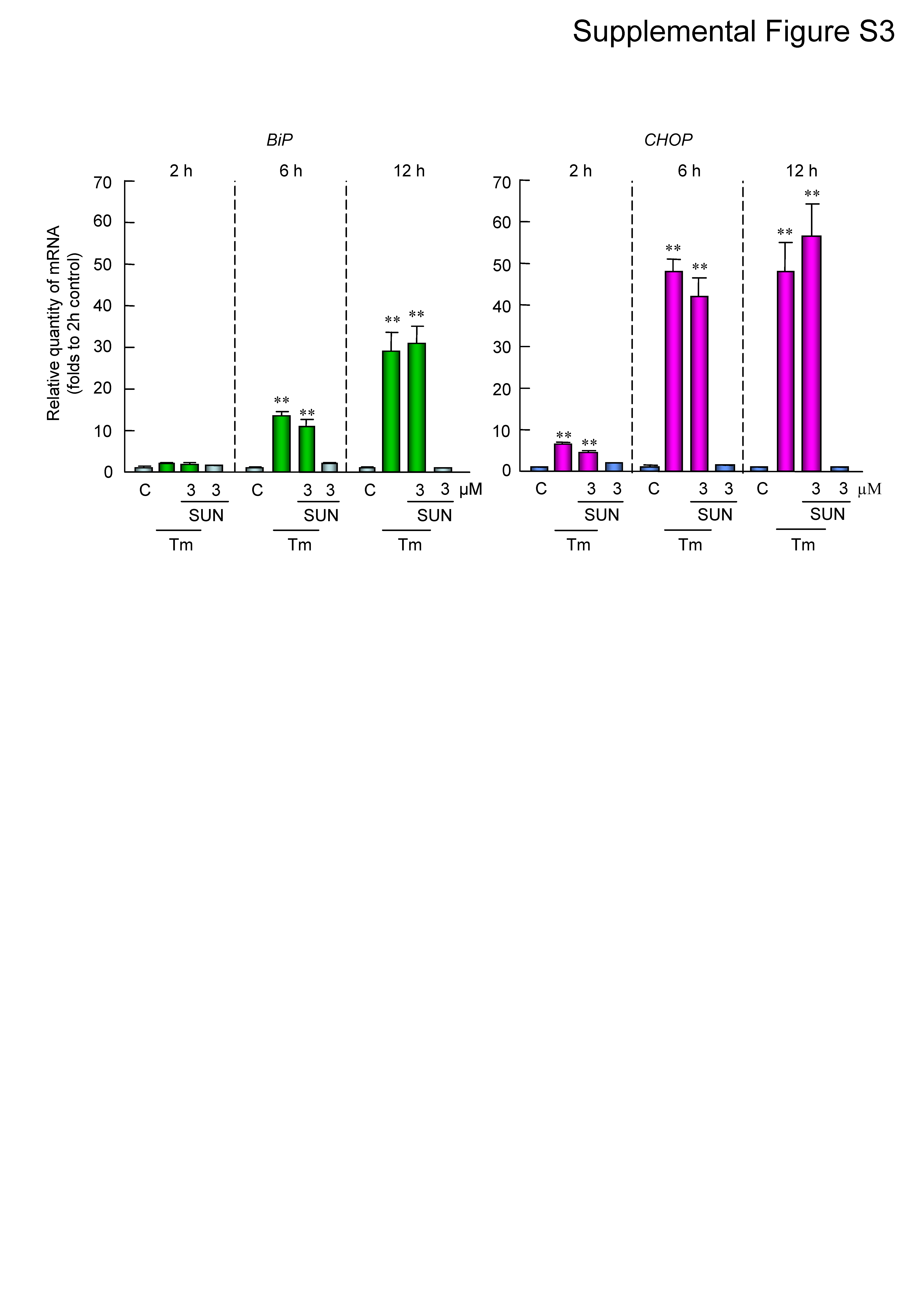

Supplement: Figure S3 — SUN N8075 does not affect gene expression level of BiP or CHOP on SH‐SY5Y cells after tunicamycin. BiP and CHOP mRNA levels were measured using a quantitative real‐time PCR. (TIF) [file pone.0015307.s004.tif]

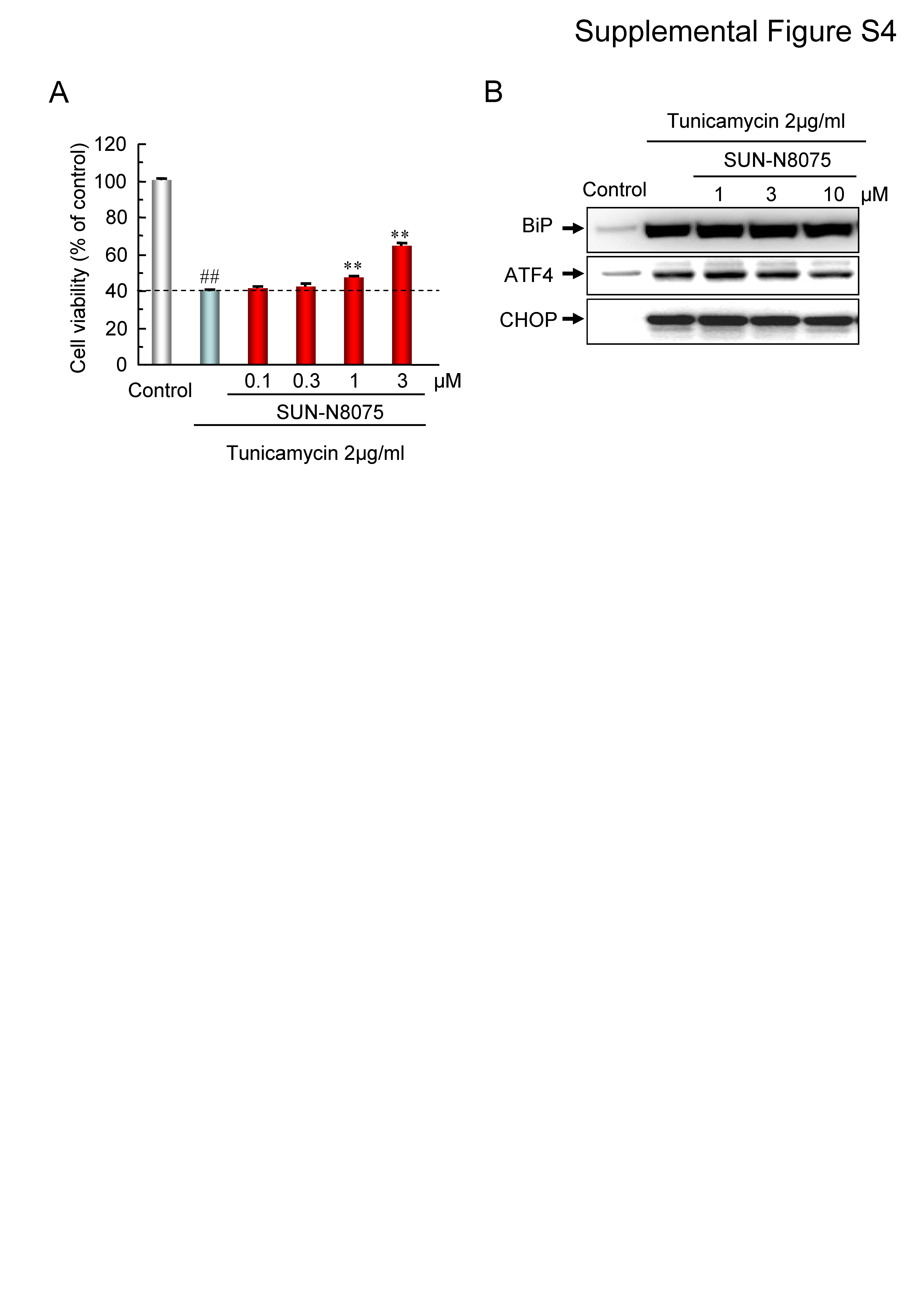

Supplement: Figure S4 — SUN N8075 protect cell death induced by tunicamycin, but did not affect protein expression of ATF4, BiP or CHOP in RGC‐5 cells. (A) SUN N8075 at 0.1 to 3 µM shows protective effect on the reduction of cell viability at 24 h after tunicamycin at 2 µg/ml. Each column represents the mean ± S.E.M. (n=8). ##p<0.01 versus control. **p<0.01 versus tunicamycin alone. (B) ATF4, BiP and CHOP protein levels were measured using Western blotting. (TIF) [file pone.0015307.s005.tif]

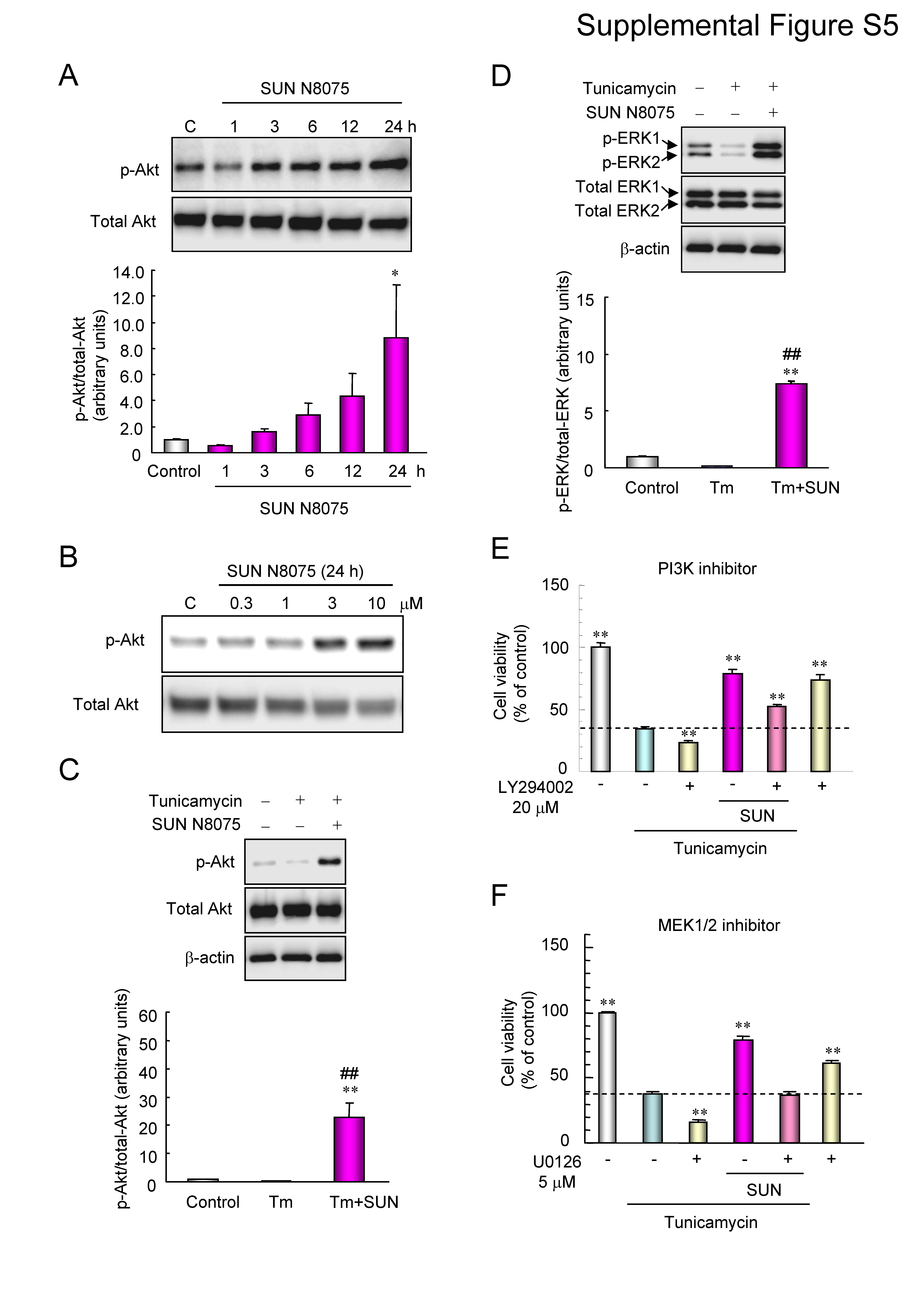

Supplement: Figure S5 — SUN N8075 enhances survival signals via Akt and ERK1/2 activations. (A) Time‐course of changes in phosphorylated‐Akt level after SUN N8075 treatment. (B) Concentration‐dependent changes in phosphorylated‐Akt level at 24 h after SUN N8075 treatment. (C, D) Tunicamycin reduced phosphorylated‐Akt (C) and phosphorylated‐ERK1/2 (D) levels, and their reductions were ameliorated by SUN N8075 treatment. (E, F) The protective effect of SUN N8075 on tunicamycin‐induced reduction of cell viability was eliminated by LY294002, a PI3kinase inhibitor, at 20 µM (E) or U0126, a MEK1/2 inhibitor, at 5 µM (F). (TIF) [file pone.0015307.s006.tif]

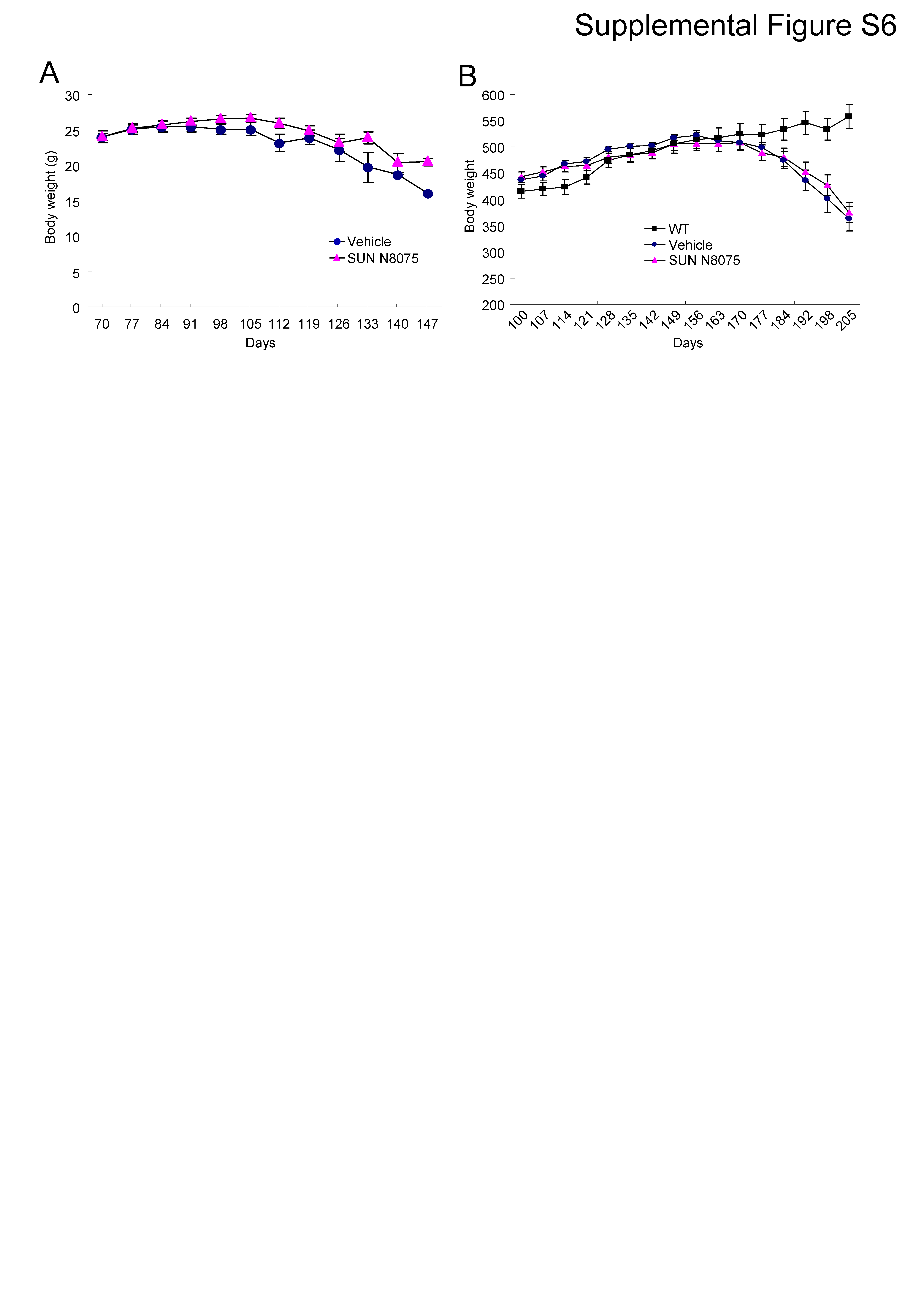

Supplement: Figure S6 — Effects of SUN N8075 on body weight changes in two type of familial ALS model animals. (A) Effect of SUN N8075 at 30 mg/kg/day, s.c. on body weight change in G93A mutant SOD1 mice. (B) Effect of SUN N8075 at 10 mg/kg/day, s.c. on body weight change in H46R mutant SOD1 rats. (TIF) [file pone.0015307.s007.tif]

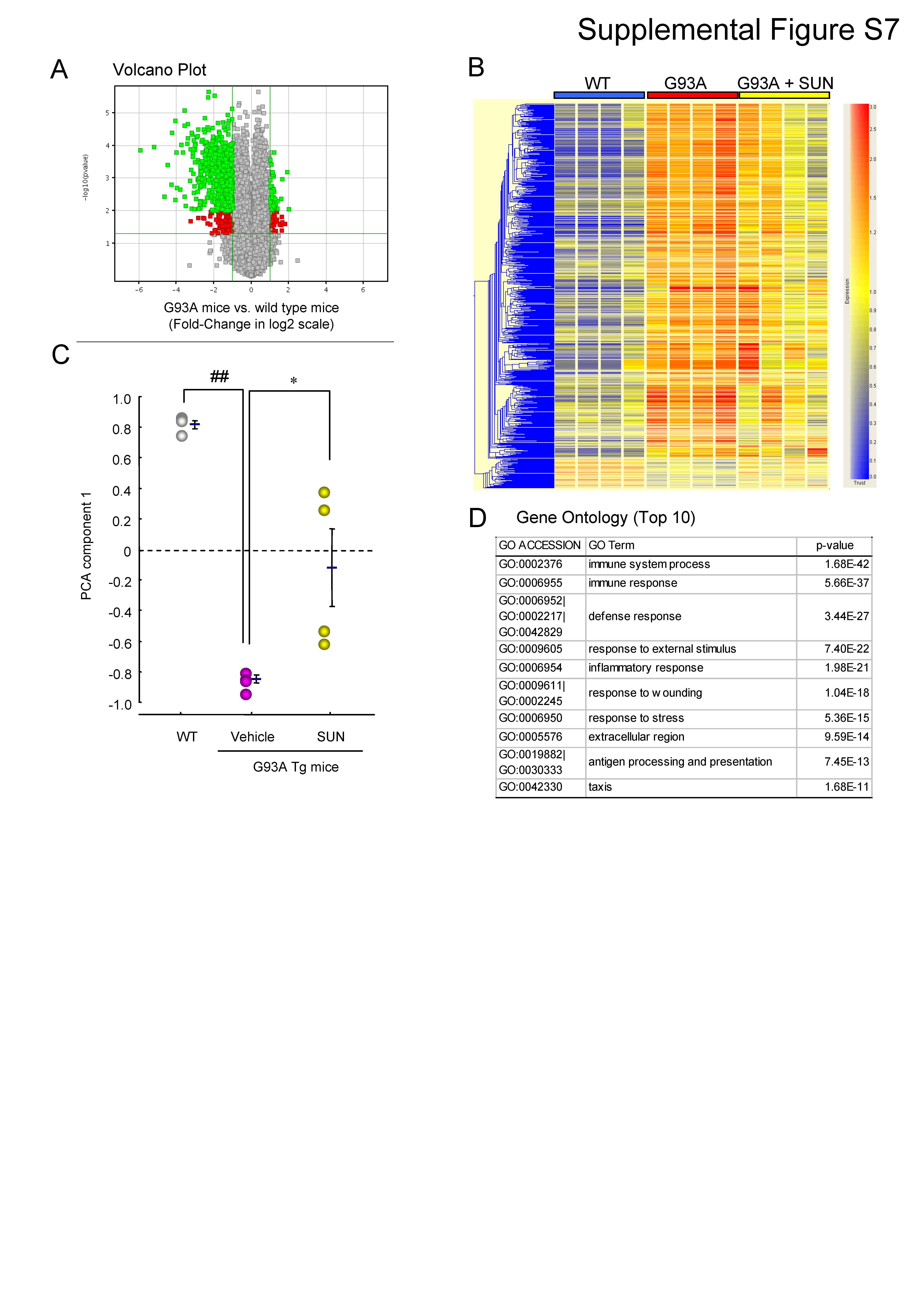

Supplement: Figure S7 — (A) Volcano plot of probe data set averages of gene expressions in the spinal cords of G93A (n=4) compared with WT (n=4). The x‐axis and y‐axis correspond to the average fold‐change (FC), and the negative log10‐transformed p value between G93A and WT mice, respectively. Green (FC ≥2, 0.01≤p≤0.05) and red (FC ≥2, p≤0.01) squares represent significantly differentially expressed probe sets, and gray squares represent probe sets with no significant difference between G93A and WT mice. The changes in gene expression were considered statistically significant based on the criteria of FC ≥2, p≤0.01 with Benjamini‐Hochberg correction. (B) Heat map representation of hierarchical clustering of 712 different gene expressions in the spinal cords of G93A compared with WT mice. The columns and rows represent the tissue samples (WT = Blue, G93A = red, G93A + SUN N8075 = yellow) and individual gene expressions, respectively. Shades of red indicate elevated expression while shades of blue indicate decreased expression relative to the median (see the color scale). (C) Principal components analysis (PCA) in the spinal cord of WT, G93A and G93A + SUN N8075 treated mice. Differently expressed 712 genes in the two groups (WT vs. G93A mice) were analyzed using a principal component analysis, and the contribution rates of genes to PCA component 1, 2, and 3 were 81.23%, 6.99%, and 3.90%, respectively. The results are expressed as a one‐dimensional function with PCA component 1 that defines the direction of greatest variation in the probe/gene transcriptomic feature space. ##p<0.01, *p<0.05, Student's t‐test. (D) Gene ontology classification for each category from the 712 different genes. (TIF) [file pone.0015307.s008.tif]
