# Supplementary material for: An Inducer of VGF Protects Cells against ER Stress-Induced Cell Death and Prolongs Survival in the Mutant SOD1 Animal Models of Familial ALS
Source: PLoS One. 2010 Dec 9;5(12):e15307. doi: 10.1371/journal.pone.0015307 (PMC3000345; doi:10.1371/journal.pone.0015307)
Supplement: Table S1 — Clinical information about the spinal cord tissues from patients with sporadic ALS (a) and for control (b). M, male; F, female; PMI, post mortem interval; y, years; m, months; h, hours. (PDF) [file pone.0015307.s009.pdf]

Supplemental Table S1.

Clinical information about the spinal cord tissues from patients with sporadic ALS (a) and for control (b).

(a) Sporadic ALS

Cases	Age of death (y)	Sex	PMI (h)	Disease duration (m)
1	59	M	3.2	48
2	83	F	2.3	8
3	57	M	2.5	9
4	76	M	1.7	22
5	80	M	2.7	72
6	73	M	*	48
Mean ± SE	71.3 ± 4.4		2.5 ± 0.2	34.5 ± 10.5

(b) Control

Cases	Age of death (y)	Sex	PMI (h)	Causes of death
1	68	M	3.8	Myopathy
2	73	M	3.3	Multiple cerebral infarction
3	64	F	2.0	Polymyositis
4	71	F	*	Myoclonus epilepsy
5	82	M	6.0	Multiple cerebral infarction
6	76	M	3.4	Multiple cerebral infarction
Mean ± SE	72.3 ± 2.5		3.7 ± 0.6	

M, male; F, female; PMI, post mortem interval; y, years; m, months; h, hours.
