# Supplementary material for: An Inducer of VGF Protects Cells against ER Stress-Induced Cell Death and Prolongs Survival in the Mutant SOD1 Animal Models of Familial ALS
Source: PLoS One. 2010 Dec 9;5(12):e15307. doi: 10.1371/journal.pone.0015307 (PMC3000345; doi:10.1371/journal.pone.0015307)
Supplement: Table S2 — Gene expression profiles after SUN N8075 treatment in SH‐SY5Y cells. (PDF) [file pone.0015307.s010.pdf]

Supplemental Table 2

Supplemental Table S2. Gene expression profiles after SUN N8075 treatment in SH-SY5Y cells

Gene name	Fold changes (vs control) 3h	7h	13h	Genbank Accession	Probe ID
PREDICTED: Homo sapiens similar to RIKEN cDNA 6332401O19 gene (LOC340344), mRNA [XM_294353	1.7	2.2	2.0	XM_294353	A_24_P695306
Unknown	1.7	2.1	2.0		A_32_P47027
Homo sapiens dickkopf homolog 1 (Xenopus laevis) (DKK1), mRNA [NM_012242	1.5	2.1	2.1	NM_012242	A_23_P24129
Homo sapiens tissue factor pathway inhibitor 2 (TFPI2), mRNA [NM_006528	1.9	2.7	2.7	NM_006528	A_23_P393620
Homo sapiens thioredoxin interacting protein (TXNIP), mRNA [NM_006472	0.6	0.5	0.5	NM_006472	A_23_P97700
Unknown	1.7	2.2	2.3		A_32_P71032
full-length cDNA clone CS0DC017YM12 of Neuroblastoma Cot 25-normalized of Homo sapiens (human). [CR59033	1.6	2.3	2.6		A_24_P33508
Homo sapiens neuronal pentraxin II (NPTX2), mRNA [NM_002523	1.0	1.3	2.2	NM_002523	A_23_P82651
Unknown	2.2	1.1	1.9		A_32_P116219
Homo sapiens layilin (LOC143903), mRNA [NM_178834	1.4	1.8	2.0	NM_178834	A_23_P127565
Homo sapiens low density lipoprotein receptor (familial hypercholesterolemia) (LDLR), mRNA [NM_00052	1.6	2.2	1.8	NM_000527	A_24_P117029
Homo sapiens HSPC053 mRNA, complete cds. [AF161538]	1.8	3.9	3.2		A_23_P140190
Homo sapiens VGF nerve growth factor inducible (VGF), mRNA [NM_003378	1.2	1.9	2.2	NM_003378	A_24_P129326
Homo sapiens ST3 beta-galactoside alpha-2,3-sialyltransferase 6 (ST3GAL6), mRNA [NM_006100	1.6	2.0	2.0	NM_006100	A_23_P250800
Homo sapiens NGFI-A binding protein 2 (EGR1 binding protein 2) (NAB2), mRNA [NM_005967	2.2	1.4	1.3	NM_005967	A_23_P76151
Homo sapiens endothelial PAS domain protein 1 (EPAS1), mRNA [NM_001430	0.7	0.4	0.3	NM_001430	A_23_P210210
Homo sapiens chromosome 21 open reading frame 93 (C21orf93), mRNA [NM_145179	1.0	0.9	2.6	NM_145179	A_24_P100190
Homo sapiens dedicator of cytokinesis 3 (DOCK3), mRNA [NM_004947	2.8	0.9	1.1	NM_004947	A_24_P272845
full-length cDNA clone CS0DM012YB14 of Fetal liver of Homo sapiens (human). [CR616003	2.8	1.1	0.3	CR616003	A_24_P65597
Homo sapiens adenosine A2a receptor (ADORA2A), mRNA [NM_000675	2.7	3.2	2.7	NM_000675	A_23_P109436
Homo sapiens growth differentiation factor 15 (GDF15), mRNA [NM_004864	2.5	2.0	2.0	NM_004864	A_23_P16523
Homo sapiens tumor necrosis factor receptor superfamily, member 25 (TNFRSF25), transcript variant 5, mRNA [NM_148968	0.8	0.5	0.4	NM_148968	A_23_P126844
full-length cDNA clone CS0DC029YA18 of Neuroblastoma Cot 25-normalized of Homo sapiens (human). [CR59356	1.5	2.0	2.6	CR593560	A_32_P219581
Homo sapiens KIAA0125 (KIAA0125), mRNA [NM_014792	1.5	4.4	3.0	NM_014792	A_24_P95723
Homo sapiens glutamate decarboxylase 1 (brain, 67kDa) (GAD1), transcript variant GAD25, mRNA [NM_013445	1.4	2.0	2.6	NM_013445	A_23_P209578
Homo sapiens 3-hydroxy-3-methylglutaryl-Coenzyme A synthase 1 (soluble) (HMGCS1), mRNA [NM_00213	1.4	2.1	1.7	NM_002130	A_24_P63522
Homo sapiens KIAA1465 protein (KIAA1465), mRNA [NM_020851	1.6	1.3	2.6	NM_020851	A_32_P379467
Homo sapiens early growth response 1 (EGR1), mRNA [NM_001964	2.7	1.9	1.7	NM_001964	A_23_P214080
Homo sapiens cDNA FLJ26323 fis, clone HRT00813, highly similar to Tissue factor pathway inhibitor 2 precursor (TFPI-2). [AK12983	2.7	3.1	2.4		A_24_P95070
full-length cDNA clone CS0DI028YI18 of Placenta Cot 25-normalized of Homo sapiens (human). [CR59315	1.1	1.6	2.1	CR593158	A_23_P112452
Homo sapiens hypothetical protein MGC39606 (MGC39606), mRNA [NM_203306	1.0	6.6	1.0	NM_203306	A_32_P104432
Homo sapiens ATPase, Na+/K+ transporting, beta 2 polypeptide (ATP1B2), mRNA [NM_001678	1.5	2.4	1.8	NM_001678	A_24_P31275
Homo sapiens calcitonin gene-related peptide-receptor component protein, mRNA (cDNA clone MGC:48670 IMAGE:5585294), complete cds. [BC04010	0.2	1.3	1.1	BC040107	A_23_P134573
Homo sapiens adenosine A2a receptor (ADORA2A), mRNA [NM_000675	3.0	3.2	2.8	NM_000675	A_24_P237270
Homo sapiens v-yes-1 Yamaguchi sarcoma viral oncogene homolog 1 (YES1), mRNA [NM_005433	0.2	1.0	1.1	NM_005433	A_24_P48403
Homo sapiens nuclear receptor subfamily 4, group A, member 1 (NR4A1), transcript variant 1, mRNA [NM_002135	2.5	1.7	1.5	NM_002135	A_23_P128230
Homo sapiens S100 calcium binding protein A16 (S100A16), mRNA [NM_080388	1.4	1.8	2.1	NM_080388	A_23_P147918
Homo sapiens phosphatidylinositol 3,4,5-trisphosphate-dependent RAC exchanger 1 (PREX1), mRNA [NM_02082	1.4	2.2	2.2	NM_020820	A_23_P413641
Unknown	2.3	1.6	1.0		A_32_P142802
Homo sapiens dihydrofolate reductase (DHFR), mRNA [NM_000791	1.1	1.1	2.7	NM_000791	A_24_P343095
Homo sapiens 24-dehydrocholesterol reductase (DHCR24), mRNA [NM_014762	1.2	2.2	1.9	NM_014762	A_23_P379475
Homo sapiens Ras-related associated with diabetes (RRAD), mRNA [NM_004165]	1.4	1.9	2.4	NM_004165	A_23_P88849
Homo sapiens phospholamban (PLN), mRNA [NM_002667]	3.3	5.4	6.6	NM_002667	A_23_P30614
Homo sapiens tumor necrosis factor receptor superfamily, member 12A (TNFRSF12A), mRNA [NM_016639]	4.2	2.1	1.9	NM_016639	A_23_P49338
Homo sapiens ankyrin repeat domain 13 family, member D (ANKRD13D), mRNA [NM_207354]	1.3	1.1	3.0	NM_207354	A_24_P76105
Homo sapiens mitogen-activated protein kinase 1 (MAPK1), transcript variant 1, mRNA [NM_002745]	0.8	1.2	2.0	NM_002745	A_24_P237265
Q9H2E3 (Q9H2E3) Neuropilin-2b(5), partial (10%) [THC2317675]	1.7	1.2	8.3		A_32_P105217
Homo sapiens neurogenin 2 (NEUROG2), mRNA [NM_024019]	0.7	0.7	0.5	NM_024019	A_32_P438767
Homo sapiens slit homolog 2 (Drosophila) (SLIT2), mRNA [NM_004787]	1.0	2.1	2.2	NM_004787	A_23_P144348
Homo sapiens sterol-C5-desaturase (ERG3 delta-5-desaturase homolog, fungal)-like (SC5DL), transcript variant 1, mRNA [NM_006918]	1.1	3.8	1.0	NM_006918	A_23_P372888
Homo sapiens heterogeneous nuclear ribonucleoprotein A1, mRNA (cDNA clone IMAGE:2900557), containing frame-shift errors. [BC010266]	0.5	0.8	1.1	BC010266	A_24_P384059
Homo sapiens low density lipoprotein receptor (familial hypercholesterolemia) (LDLR), mRNA [NM_000527]	1.6	2.3	1.5	NM_000527	A_23_P208595
Homo sapiens natriuretic peptide precursor A (NPPA), mRNA [NM_006172]	0.8	0.5	0.5	NM_006172	A_23_P74059
Homo sapiens fibronectin leucine rich transmembrane protein 3 (FLRT3), transcript variant 2, mRNA [NM_198391]	0.5	0.7	0.5	NM_198391	A_23_P166109
Homo sapiens cDNA clone IMAGE:30325817. [BC092452]	1.5	1.1	0.4	BC092452	A_24_P548415
Homo sapiens angiotensinogen (serine (or cysteine) proteinase inhibitor, clade A (alpha-1 antiproteinase, antitrypsin), member 8) (AGT), mRNA [NM_000029]	1.2	1.4	2.1	NM_000029	A_23_P115261
Homo sapiens family with sequence similarity 84, member A (FAM84A), mRNA [NM_145175]	0.8	0.7	0.5	NM_145175	A_23_P306215
Homo sapiens protein tyrosine phosphatase, receptor type, E (PTPRE), transcript variant 1, mRNA [NM_006504]	1.8	2.1	1.8	NM_006504	A_24_P213503

Supplemental Table 2

Homo sapiens BAI1-associated protein 2 (BAIAP2), transcript variant 3, mRNA [NM_006340]	1.0	1.0	0.5	NM_006340	A_24_P159648
Homo sapiens dehydrogenase/reductase (SDR family) member 3 (DHRS3), mRNA [NM_004753]	0.8	0.4	0.3	NM_004753	A_23_P33759
Homo sapiens NGFI-A binding protein 2 (EGR1 binding protein 2) (NAB2), mRNA [NM_005967]	2.2	1.5	1.3	NM_005967	A_23_P368187
AW268902 xv48h10.x1 Soares_NFL_T_GBC_S1 Homo sapiens cDNA clone IMAGE:2816419 3', mRNA sequence [AW268902]	1.1	2.5	2.3	AW268902	A_32_P106615
Homo sapiens inhibitor of DNA binding 4, dominant negative helix-loop-helix protein (ID4), mRNA [NM_001546]	2.7	2.3	1.7	NM_001546	A_23_P59375
Homo sapiens intercellular adhesion molecule 5, telencephalin (ICAM5), mRNA [NM_003259]	9.9	3.1	1.1	NM_003259	A_23_P119143
Homo sapiens hypothetical protein FLJ37266 (FLJ37266), mRNA [NM_175892]	0.9	0.6	0.4	NM_175892	A_32_P229618
Homo sapiens cellular retinoic acid binding protein 2 (CRABP2), mRNA [NM_001878]	1.0	0.7	0.4	NM_001878	A_23_P115064
Homo sapiens testis-specific kinase 1 (TESK1), mRNA [NM_006285]	1.2	1.2	0.2	NM_006285	A_24_P341882
PREDICTED: Homo sapiens similar to peptidyl-Pro cis trans isomerase (LOC392352), mRNA [XM_373301]	0.9	0.4	1.1	XM_373301	A_24_P401036
Homo sapiens dipeptidylpeptidase 9 (DPP9), mRNA [NM_139159]	1.0	1.1	0.0	NM_139159	A_23_P208915
Homo sapiens cDNA: FLJ22798 fis, clone KAIA2617. [AK026451]	0.9	1.0	0.5	AK026451	A_23_P358221
	0.4	1.0	1.0		A_24_P685241
Homo sapiens sulfatase 2 (SULF2), transcript variant 1, mRNA [NM_018837]	1.0	0.6	0.4	NM_018837	A_23_P154605
Homo sapiens zinc finger protein 429 (ZNF429), mRNA [NM_001001415]	2.6	1.4	0.9	NM_001001415	A_32_P106944
Homo sapiens PDZ domain containing RING finger 3 (PDZRN3), mRNA [NM_015009]	0.5	0.4	0.3	NM_015009	A_23_P21618
Homo sapiens angiopoietin-like 4 (ANGPTL4), transcript variant 2, mRNA [NM_016109]	0.7	0.2	0.3	NM_016109	A_23_P159325
Homo sapiens cDNA FLJ46080 fis, clone TESTI2004971. [AK127966]	1.0	1.0	0.5	AK127966	A_24_P920278
Homo sapiens Rho GDP dissociation inhibitor (GDI) beta (ARHGDIB), mRNA [NM_001175]	1.3	1.4	2.0	NM_001175	A_23_P151075
601854749F1 NIH_MGC_57 Homo sapiens cDNA clone IMAGE:4074517 5', mRNA sequence [BF246504]	1.4	0.9	0.5	BF246504	A_24_P612200
Homo sapiens full length insert cDNA clone ZE12B03. [AF086547]	0.7	0.6	0.5	AF086547	A_32_P203749
Homo sapiens arrestin, beta 1 (ARRB1), transcript variant 1, mRNA [NM_004041]	1.2	1.1	0.5	NM_004041	A_24_P386622
Homo sapiens UDP-N-acetyl-alpha-D-galactosamine:polypeptide N-acetylgalactosaminyltransferase-like 4 (GALNTL4), mRNA [NM_198516]	1.0	0.7	0.4	NM_198516	A_23_P139418
Homo sapiens ORM1-like 3 (S. cerevisiae) (ORMDL3), mRNA [NM_139280]	0.4	0.9	1.0	NM_139280	A_23_P38190
	1.1	1.1	0.0		A_32_P200773
AI857589 wk95b07.x1 NCI_CGAP_Lu19 Homo sapiens cDNA clone IMAGE:2423125 3' similar to contains Alu repetitive element;contains element MER22 repetitive element ;, mRNA sequence [AI857589]	1.0	0.4	1.0	AI857589	A_32_P9816
Homo sapiens growth differentiation factor 10 (GDF10), mRNA [NM_004962]	3.3	0.8	0.3	NM_004962	A_23_P52227
full-length cDNA clone CL0BB018ZH05 of Neuroblastoma of Homo sapiens (human). [CR618615]	2.1	1.3	1.1	CR618615	A_32_P8546
Homo sapiens inositol polyphosphate-5-phosphatase, 75kDa (INPP5B), mRNA [NM_005540]	0.3	0.8	0.8	NM_005540	A_23_P85640
Homo sapiens cDNA FLJ14704 fis, clone NT2RP3000526. [AK027610]	1.3	1.0	0.5	AK027610	A_24_P937582
Homo sapiens family with sequence similarity 22, member A, mRNA (cDNA clone IMAGE:5258548), partial cds. [BC033231]	1.5	0.7	0.5		A_32_P799227
Homo sapiens cDNA FLJ31715 fis, clone NT2RI2006553. [AK056277]	0.8	0.9	0.4	AK056277	A_23_P343330
Homo sapiens mRNA for KIAA1183 protein, partial cds. [AB033009]	0.4	0.9	0.8	AB033009	A_23_P383101
Q6E5T4 (Q6E5T4) Claudin 2, partial (5%) [THC2343678]	0.5	0.5	0.6		A_32_P38093
Homo sapiens chromosome 9 open reading frame 100 (C9orf100), mRNA [NM_032818]	0.8	0.8	0.4	NM_032818	A_24_P30034
Homo sapiens purinergic receptor P2Y, G-protein coupled, 11 (P2RY11), mRNA [NM_002566]	0.9	1.0	0.3	NM_002566	A_23_P4696
Homo sapiens GRIP and coiled-coil domain containing 2 (GCC2), transcript variant 1, mRNA [NM_181453]	0.8	1.1	0.3	NM_181453	A_24_P290502
	1.0	1.1	0.3		A_24_P84608
Homo sapiens Kruppel-like factor 2 (lung) (KLF2), mRNA [NM_016270]	0.3	1.0	0.7	NM_016270	A_24_P151305
Homo sapiens ring finger protein 113B (RNF113B), mRNA [NM_178861]	0.9	1.0	0.3	NM_178861	A_24_P297480
	1.0	0.9	0.2		A_24_P170365
STPHRG deoxyribodipyrimidine photolyase {Salmonella typhimurium;} , partial (4%) [THC2388093]	0.9	0.9	0.3		A_32_P186348
Homo sapiens cDNA FLJ30901 fis, clone FEBRA2005778, weakly similar to INTEGUMENTARY MUCIN A.1 PRECURSOR. [AK055463]	0.9	0.7	0.4	AK055463	A_23_P343104
Homo sapiens cDNA FLJ11561 fis, clone HEMBA1003142. [AK021623]	0.2	0.8	0.9	AK021623	A_32_P1533
	0.8	0.2	0.2		A_23_P170719
Homo sapiens hypothetical protein FLJ12476 (FLJ12476), mRNA [NM_022784]	1.2	0.7	0.2	NM_022784	A_24_P275585
UI-E-DX0-ago-d-21-0-UI.s1 UI-E-DX0 Homo sapiens cDNA clone UI-E-DX0-ago-d-21-0-UI 3', mRNA sequence [BM667062]	1.1	1.1	0.2	BM667062	A_32_P164225
BC064838 makorin, ring finger protein, 1 {Homo sapiens;} , partial (61%) [THC2312236]	1.1	1.2	0.2		A_24_P281844
Homo sapiens Ras-associated protein Rap1 (RBJ), mRNA [NM_016544]	1.1	0.9	0.2	NM_016544	A_23_P17275
	0.2	0.9	1.0		A_24_P93012
Homo sapiens Ras association (RalGDS/AF-6) domain family 2 (RASSF2), transcript variant 2, mRNA [NM_170773]	1.0	32.2	1.1	NM_170773	A_24_P288448
Homo sapiens Down syndrome cell adhesion molecule like 1 (DSCAML1), mRNA [NM_020693]	0.5	162.5	0.8	NM_020693	A_23_P47340
Homo sapiens crystallin, gamma C (CRYGC), mRNA [NM_020989]	0.8	0.6	0.1	NM_020989	A_23_P142606
BCR...ABL {b3/a3 junction, translocation breakpoint} [human, Japanese CML patient 1 and ALL patient 2, peripheral blood, mononuclear cells, mRNA Mutant, 3 genes, 140 nt]. [S72478]	1.1	1.0	0.1	S72478	A_24_P922271
