# Supplementary material for: An Inducer of VGF Protects Cells against ER Stress-Induced Cell Death and Prolongs Survival in the Mutant SOD1 Animal Models of Familial ALS
Source: PLoS One. 2010 Dec 9;5(12):e15307. doi: 10.1371/journal.pone.0015307 (PMC3000345; doi:10.1371/journal.pone.0015307)
Supplement: Table S3 — Gene expression profiles for ER‐stress responsible genes after SUN N8075 alone, or tunicamycin with or without SUN N8075 in SH‐SY5Y cells. ↑: two‐fold or more increase, →: no changes. (PDF) [file pone.0015307.s011.pdf]

Supplemental Table 3

Supplemental Table S3. Gene expression profiles for ER-stress responsible genes after SUN N8075 alone, or tunicamycin with or without SUN N8075 in SH-SY5Y cells.

	Gene Symbol	Genbank Accession	Fold change (vs. control)						
			Time after SUN N8075			6 h after Tm		12 h after Tm	
						Vehicle	SUN	Vehicle	SUN
			3 h	7 h	13 h	7 h	7 h	13 h	13 h
CEBPG: CCAAT/enhancer binding protein (C/EBP), gamma	CEBPG	NM_001806	1.12 →	1.04 →	1.16 →	4.31 ↑	4.80 ↑	6.13 ↑	7.01 ↑
Activating transcription factor 3	ATF3	NM_001040619	1.25 →	1.31 →	1.18 →	13.31 ↑	17.21 ↑	6.99 ↑	9.32 ↑
Homocysteine-inducible, endoplasmic reticulum stress-inducible, ubiquitin-like domain member 1	HERPUD	NM_014685	0.96 →	0.99 →	1.00 →	4.67 ↑	4.52 ↑	10.84 ↑	11.31 ↑
CCAAT/enhancer binding protein (C/EBP), epsilon	CEBPE	NM_001805	1.24 →	1.33 →	1.20 →	7.76 ↑	8.68 ↑	9.95 ↑	11.42 ↑
CCAAT/enhancer binding protein (C/EBP), beta	CEBPB	NM_005194	1.36 →	1.43 →	1.50 →	10.32 ↑	11.36 ↑	15.48 ↑	17.20 ↑
Heat shock 70kDa protein 5 (glucose-regulated protein, 78kDa)	HSPA5	NM_005347	1.03 →	1.20 →	1.09 →	5.85 ↑	4.78 ↑	21.65 ↑	19.30 ↑
DNA-damage-inducible transcript 3 (GADD153)	DDIT3	NM_004083	0.95 →	1.08 →	1.16 →	18.99 ↑	20.24 ↑	34.09 ↑	40.99 ↑
Growth arrest and DNA-damage-inducible, beta	GADD45	NM_015675	1.00 →	1.10 →	0.68 →	2.47 ↑	2.09 ↑	1.27 →	0.77 →
CCAAT/enhancer binding protein (C/EBP), delta	CEBPD	NM_005195	1.56 →	1.44 →	1.34 →	2.44 ↑	3.63 ↑	1.66 →	2.34 ↑
Activating transcription factor 4 (tax-responsive enhancer element B67)	ATF4	NM_001675	1.09 →	0.98 →	1.12 →	2.44 ↑	2.28 ↑	3.13 ↑	3.07 ↑
DNA-damage-inducible transcript 4	DDIT4	NM_019058	0.96 →	0.93 →	1.02 →	4.47 ↑	4.73 ↑	3.62 ↑	4.14 ↑
Heat shock protein 90kDa beta (Grp94), member 1	HSP90B	NM_003299	1.03 →	1.15 →	0.96 →	2.10 ↑	2.20 ↑	3.94 ↑	4.27 ↑
X-box binding protein	XBP1	NM_005080	1.22 →	1.24 →	1.23 →	5.54 ↑	6.05 ↑	8.21 ↑	8.67 ↑
Tribbles homolog 3 (Drosophila)	TRIB3	NM_021158	1.37 →	1.09 →	1.04 →	8.77 ↑	9.66 ↑	14.27 ↑	15.49 ↑
ER degradation enhancer, mannosidase alpha-like 1	EDEM1	NM_014674	1.59 →	1.73 →	1.34 →	1.78 →	2.21 ↑	5.12 ↑	6.21 ↑

↑ : two-fold or more increase, →: no changes
