# Supplementary material for: An Inducer of VGF Protects Cells against ER Stress-Induced Cell Death and Prolongs Survival in the Mutant SOD1 Animal Models of Familial ALS
Source: PLoS One. 2010 Dec 9;5(12):e15307. doi: 10.1371/journal.pone.0015307 (PMC3000345; doi:10.1371/journal.pone.0015307)
Supplement: Table S4 — SUN N8075 prolongs disease onset and lifspan in a rat model of familial ALS. Data are the means ± S.E. (n=9 or 10), * P<0.05 vs vehicle‐treated ALS mouse group. a Observable functional deficits (motor score of 4), b Righting reflex failure (motor score of 1), c Motor score of 0. (PDF) [file pone.0015307.s012.pdf]

Supplemental Table 4

Supplemental Table S4. SUN N8075 prolongs disease onset and lifspan in a rat model of familial ALS.

	H46R rat	
	Vehicle	SUN N8075
Oneset[a]	175.6 ± 3.4	187.6 ± 3.4 *
Endostage[b]	199.6 ± 4.4	212.1 ± 2.6 *
Lifespan[c]	201.1 ± 4.7	214.7 ± 2.0 *

Data are the means ± S.E. (*n* = 9 or 10), * *P* < 0.05 vs vehicle-treated ALS mouse group. A Observable functional deficits (motor score of 4), b Righting reflex failure (motor score of 1), c Motor score of 0.
